# ORC1 binds to *cis*-transcribed RNAs for efficient activation of replication origins

**DOI:** 10.1038/s41467-023-40105-3

**Published:** 2023-07-24

**Authors:** Aina Maria Mas, Enrique Goñi, Igor Ruiz de los Mozos, Aida Arcas, Luisa Statello, Jovanna González, Lorea Blázquez, Wei Ting Chelsea Lee, Dipika Gupta, Álvaro Sejas, Shoko Hoshina, Alexandros Armaos, Gian Gaetano Tartaglia, Shou Waga, Jernej Ule, Eli Rothenberg, María Gómez, Maite Huarte

**Affiliations:** 1 Center for Applied Medical Research, University of Navarra, Pio XII 55 Ave, 31008 Pamplona, Spain; 2 Institute of Health Research of Navarra (IdiSNA), Cancer Center Clínica Universidad de Navarra (CCUN), Pamplona, Spain; 3 RNA Networks Lab, The Francis Crick Institute, NW11BF London, UK; 4 Department of Biochemistry and Molecular Pharmacology, Perlmutter Cancer Center, New York University School of Medicine, New York, NY 10016 USA; 5 Department of Chemical and Biological Sciences, Japan Women’s University, Tokyo, 112-8681 Japan; 6 Center for Human Technologies, Istituto Italiano di Tecnologia, Genova, Italy; 7 Centre for Genomic Regulation (CRG), The Barcelona Institute for Science and Technology, Universitat Pompeu Fabra (UPF), Barcelona, Spain; 8 Department of Biology ‘Charles Darwin’, Sapienza University of Rome, Rome, Italy; 9 Institució Catalana de Recerca i Estudis Avançats (ICREA), Barcelona, Spain; 10 Centro de Biología Molecular Severo Ochoa (CBMSO), Consejo Superior de Investigaciones Científicas/Universidad Autónoma de Madrid (CSIC/UAM), Nicolás Cabrera 1, 28049 Madrid, Spain; 11 Present Address: Neurosciences Area, Biodonostia Health Research Institute, 20014 San Sebastian, Spain; 12 Present Address: Ikerbasque, Basque Foundation for Science, 48009 Bilbao, Spain

**Keywords:** Molecular biology, Non-coding RNAs, Origin firing

## Abstract

Cells must coordinate the activation of thousands of replication origins dispersed throughout their genome. Active transcription is known to favor the formation of mammalian origins, although the role that RNA plays in this process remains unclear. We show that the ORC1 subunit of the human Origin Recognition Complex interacts with RNAs transcribed from genes with origins in their transcription start sites (TSSs), displaying a positive correlation between RNA binding and origin activity. RNA depletion, or the use of ORC1 RNA-binding mutant, result in inefficient activation of proximal origins, linked to impaired ORC1 chromatin release. ORC1 RNA binding activity resides in its intrinsically disordered region, involved in intra- and inter-molecular interactions, regulation by phosphorylation, and phase-separation. We show that RNA binding favors ORC1 chromatin release, by regulating its phosphorylation and subsequent degradation. Our results unveil a non-coding function of RNA as a dynamic component of the chromatin, orchestrating the activation of replication origins.

## Abstract (web summary)

Here the authors describe that the human origin recognition complex subunit 1 (ORC1) binds to RNAs transcribed from genes with origins of replication at their TSS impacting origin activation.

## Introduction

The initiation of DNA replication involves a sequential assembly and disassembly of protein complexes on genomic DNA, which is tightly controlled along the cell cycle. Initiation occurs at specific sites throughout the genome where the Origin Recognition Complex (ORC) associates in M/G1. ORC is composed of six subunits (ORC1-6), of which ORC1 is the pioneering subunit in the binding to the chromatin. This binding recruits other members of the complex, followed by additional initiation factors (CDC6, CDT1, MCM helicases, CDC45, and CDC7) in a sequential manner, leading to, (i) licensing and (ii) firing of replication origins^1^. Linked to the firing in the S phase, some components of the initiation complex are disassembled and targeted for degradation, which is followed by the activation of DNA helicases and loading of replication factors, avoiding DNA re-duplication events in the same cycle^2,3^. ORC1 stands out among ORC components in its distinct regulation throughout the cell cycle, which is consistent with its crucial function in the initiation of replication and the maintenance of undamaged cell propagation^2,4^.

In *S. cerevisiae*, the position of replication origins is defined by ORC recognition of DNA sequence-dependent elements^5^. In contrast, how origins are positioned in mammalian genomes is still an outstanding question, since ORC does not recognize a known consensus DNA sequence^6^. Furthermore, only ~20% of licensed origins in a given cell fire in the S phase^7^, but how this activation is regulated is not fully understood. Multiple chromatin features are known to influence the flexible selection and activation of origins, which, in combination, dictate the probability of stochastic origin activation. These include chromatin accessibility, specific histone marks such as H4K20me2^8,9^ and H2AZ^10^, and the presence of DNA sequences prone to form G-quadruplexes (G4)^11–13^. While the simultaneous replication and transcription of a precise DNA position are strictly incompatible, the most active origins are localized at transcription start sites (TSSs), with origin activity correlating with the level of gene expression^14–20^. Thus, in the entry of the S phase, when early replication origins are fired, RNA is produced in close proximity. This raises the possibility that RNAs could influence origin selection or activation. While previous reports have pointed to specific roles for RNA in replication initiation in *X. laevis* shortly after fertilization^21^ and at Epstein Barr virus OriP^22^, whether transcribed RNA plays a general role in the activation of mammalian origins yet remains unknown.

Here, we show that ORC1 interacts with RNAs transcribed at active origins, which is linked to ORC1 dynamic association with the chromatin and has an impact on replication origin activity.

## Results

### ORC1 interacts with RNA in vivo

**Fig. 1:**
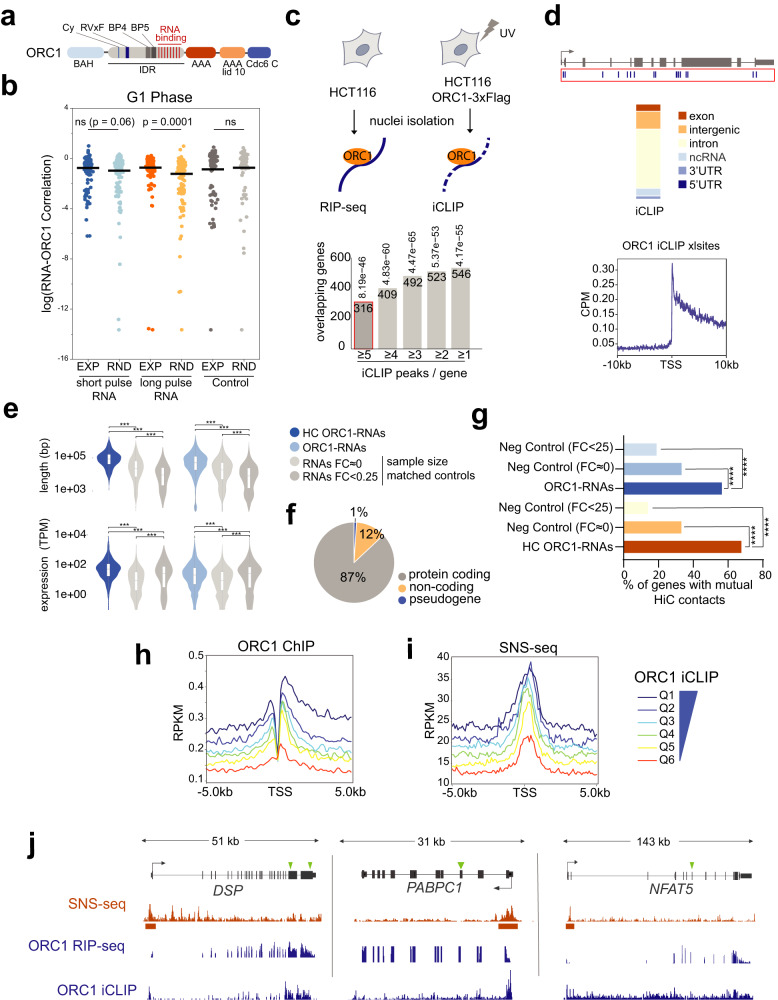
ORC1 binds in vivo to RNAs produced from active replication origins. **a** Schematic representation of human ORC1 protein domains^4,24,103^. **b** Cross-correlation between endogenous ORC1, and unlabeled (control) or EU-labeled RNA (short or long pulse), comparing STORM experimental (EXP) and randomized (RND) analysis in the chromatin fraction of U2OS cells synchronized in G1. Data were presented as mean values (*n* > 50 cells). Indicated *p* values (ns denoting *p* value >0.05) derive from unpaired two-sample *t*-test. **c** Schematic of RIP-seq and iCLIP experimental approaches, where endogenous or Flag-tagged ORC1 is immunoprecipitated from native or UV-crosslinked nuclear extracts, followed by recovery of full-length or digested bound RNAs. Below, the number of genes identified by both methods, with different iCLIP stringencies, and hypergeometric test *p* values of the experimental overlap (RIP-iCLIP) on top of the bars; red for selected high confidence (HC) ORC1-RNAs. **d** Genomic distributions of ORC1 iCLIP crosslinks, and (below) their density around TSSs (−/+ 10 kb) of ORC1-bound genes. **e** Gene length and expression level of high confidence (HC) ORC1-RNAs (iCLIP-RIP overlap) and ORC1-RNAs (iCLIP-RIP union), relative to sample size-matched control genes with different fold changes (FC) in ORC1 RIP-seq. *n* = number of genes in each category (Supplementary Data 2; from iCLIP data [>5 crosslink sites and <0.05 FDR] and RIP-seq data [log2 fold change >1 and *p* value <0.05]). Box plots show the median distribution between Q1 and Q3. *** denotes *p* value <0.001, derived from unpaired two-tailed Student’s *t*-test. **f** Gene biotypes of ORC1-RNAs. **g** Percentage of ORC1-RNA and high confidence ORC1-RNA (HC) genes with mutual interactions according to Hi-C analysis, compared to controls shown in Fig. 1e. Bars represent mean values. **** denotes *p* value <0.0001, derived from a two-proportion *z*-test. **h**, **i** Density plots of **h** ORC1 ChIP-seq and **i** SNS-seq normalized reads across six quantiles (Q) of ORC1-RNA genes, defined by ORC1 iCLIP, centered around their TSSs (−/+ 5 kb). **j** Browser snapshot of representative high confidence ORC1-RNA genes, showing data of ORC1 RNA-binding (ORC1 RIP-seq or iCLIP crosslinks), and replication origins (SNS-seq) at their TSSs, in HCT116 cells. Green arrows indicate positions of GAA repeats.

We hypothesized that ORC1, the first subunit of the initiation complex associated with the chromatin, binds to RNA in cells. In vitro RNA binding had been described for human ORC1, mapping to a region between amino acids 413 and 511^23,24^, part of an Intrinsically Disordered Region (IDR) and separated from other domains mediating, among other functions, its binding to nucleosomes^4,25^ (Fig. 1a and Supplementary Fig. 1a). In line with this hypothesis, stochastic optical reconstruction microscopy (STORM) on the chromatin fraction of G1-synchronized cells detected a significant cross-correlation between ORC1 and EU-labeled RNA, when compared to randomized images, and not detected in the experimental negative control (Fig. 1b and Supplementary Fig. 1b). These results indicate that ORC1 is in very close proximity to RNA in vivo.

We then set out to identify RNAs bound by ORC1 by applying complementary approaches (Fig. 1c). First, we performed native ORC1 RNA immunoprecipitation coupled to sequencing (RIP-seq) of total RNA (ribo-depleted polyA+ and polyA- RNA) from nuclei of dividing HCT116 cells (Supplementary Fig. 2a, b), using an anti-ORC1 antibody. These experiments identified 2203 RNAs enriched in ORC1 RIP relative to the input samples of nuclear RNA (Log2 fold change >1, *p* value <0.05), while the control IgG did not recover detectable amounts of RNA. To confirm the capacity of immunoprecipitated ORC1 to retrieve RNA, we also performed anti-Flag RIP-seq from nuclear extracts of HCT116 cells transiently expressing ORC1-3xFlag. Most of the identified transcripts (68%) overlapped with the ones interacting with the endogenous ORC1 (Hypergeometric test, *p* value 1.89e-191) (Supplementary Data 1). Next, theoretical binding predictions were calculated to examine the plausibility of direct interaction between ORC1 and the co-immunoprecipitated RNAs. *cat*RAPID algorithm, which computes an interaction probability based on the biophysical characteristics of proteins and RNAs^26^, showed that enriched RNAs by RIP also present higher predicted binding compared to the depleted ones (*p* value <2.2 e^−16^). Moreover, it showed a correlation between theoretical binding score and experimental fold changes in RIP-seq (Supplementary Fig. 2c, d), thus suggesting that direct binding occurs between the experimentally-defined set of RNAs and ORC1.

We therefore set to detect the direct RNA-protein interactions with nucleotide resolution by applying the iCLIP protocol^27^. HCT116 cells expressing ORC1-3xFlag were UV-crosslinked prior to nuclear isolation and anti-Flag immunoprecipitation. Affinity-purified complexes were partially RNase digested, and protected RNAs at and above ORC1-expected molecular weight were extracted, sequenced, and compared with the negative control (i.e., anti-Flag IP in cells that do not express ORC1-3xFlag), which only recovered neglectable RNA amounts (Fig. 1c, Supplementary Fig. 2e, and Supplementary Table 1). Read alignment to the human genome identified the vast majority of ORC1 crosslink RNA-binding sites (>95%) covering genic regions on the same direction of transcription. While ORC1-RNA crosslinks peaked at the 5’ of genes, they mapped to both exonic and intronic regions, indicating binding to nascent transcripts (Fig. 1d and Supplementary Fig. 2f).

Together, these results demonstrate that ORC1 interacts with RNA in the nucleus of living cells, which could shape protein functionality along the cell cycle.

### ORC1 binds GAA-rich and highly transcribed RNAs, sequence-independently

Once established that ORC1 interacts with RNA in the nucleus of cells, we next explored whether it prefers to associate through specific RNA motifs. To do that, we searched for motifs in 200-nt windows centered around the iCLIP peaks defined with iCOUNT peak caller. This analysis identified the snoRNA C box UGAUGA motif (*e-*value 6.0e-246) (Supplementary Fig. 2g), in line with the presence of a high number of crosslinks (35%) corresponding to highly expressed snoRNAs, although only mapping to 6% of the genes of ORC1-bound RNAs (Supplementary Data 2). Consistently, when snoRNAs were filtered out, no motif was found enriched, indicating that the binding of ORC1 to the majority of RNAs (94% of genes) is not mediated through a well-defined sequence. Since ORC1 had been reported to preferentially bind to G-quadruplex RNA structures^24,28^, we also performed G4 predictions around iCLIP peaks, which found a mild enrichment of RNA sequences prone to form this type of secondary structures (Supplementary Fig. 2h). These analyses suggest that ORC1 does not bind to RNA through a specific sequence, although it shows some preference toward structured RNA elements.

To understand the nature of ORC1 RNA interactome, we compared the results of ORC1 RIP-seq and iCLIP experiments. Interestingly, RIP-seq and iCLIP showed good agreement, since ORC1 RNA interactors identified by iCLIP also showed higher fold enrichments by RIP (Supplementary Fig. 2i), even when RIP interactors are defined relative to their input, and therefore not biased by expression level. Moreover, by astringently selecting RNAs containing 5 or more iCLIP peaks (1887 genes), a significant overlap (Hypergeometric test, *p* value 8,19e^−46^) was found with the RNAs identified by RIP-seq (Fig. 1c), representing high confidence RNAs directly interacting with ORC1 (HC ORC1-RNAs) (Supplementary Data 2). When the selection was extended to genes with more than 4, 3, or 2 iCLIP peaks, the overlap was increased and also highly significant (Fig. 1c). In addition, several RNA interactors detected by RIP-seq and/or iCLIP were validated in independent RIP and CLIP experiments, with no enrichment detected for the unspecific IgG (Supplementary Fig. 2j, k), supporting the validity of the used complementary analyses (Supplementary Fig. 2l).

We then looked at the characteristics of both the overlapping and the broader set of identified RNAs. HC ORC1-RNAs, as well as the larger set of ORC1-RNAs (Supplementary Data 2), are mostly mRNAs (95% and 87%), with higher expression levels and length than negative control genes (Fig. 1e, f). Similar features were found when assessing RIP-RNAs or iCLIP-RNAs separately (Supplementary Fig. 3a, b), confirming that ORC1 preferentially binds to RNAs of these characteristics in physiological conditions. According to Repli-seq and Hi-C analyses, the majority of genes encoding for HC ORC1-RNAs and ORC1-RNAs are localized within the early-replicating regions of the genome (80%, *p* value 4e^−253^ and 66.47%, *p* value 2e^−164^), and interact with each other in the 3D nucleus with higher frequency (*p* value <2.2e^−16^ for both sets, Fig. 1g). Interestingly, sequence analyses performed on full-length HC ORC1-RNAs showed the enrichment of tandem GAA repeats (*e*-value 7.5e^−11^, Supplementary Fig. 3c), known to be linked to nuclear retention of certain mRNAs^29^. The sequences were also highly enriched in RNAs detected by RIP-seq of endogenous ORC1 and ORC1-3xFlag, with 82 and 94% of the RNAs containing this type of sequence, respectively (Supplementary Fig. 3d, e). However, the position of iCLIP peaks indicates that ORC1 does not bind RNA through it, as confirmed by the similar EMSA behavior of ORC1-RNAs regardless of the presence of this sequence (Supplementary Fig. 3f–h). We then concluded that the presence of this motif represents a feature common to many of the RNAs bound by ORC1.

In summary, ORC1 binds to long and highly expressed nuclear RNAs. While this binding appears to be sequence-independent, ORC1-RNA interactors show distinctive features, such as the presence of GAA repeats and their production from genes that replicate in the early S phase and are in 3D proximity.

### RNAs interacting with ORC1 are transcribed from active origins

Since efficient origins are frequently localized near transcription start sites of highly transcribed genes^14–20^, we hypothesized that the binding of ORC1 to RNA could take place in the proximity of the loci where the RNAs are produced. We investigated the chromatin at the TSSs of the genes encoding for ORC1-RNAs, by grouping them in 6 quantiles according to their level of ORC1 iCLIP signal, that is, the level of direct ORC1-RNA interaction determined experimentally. This analysis showed a positive correlation between ORC1 binding to RNAs, marks of active chromatin (H3K27ac, H3K4me1, H3K4me3, and H3K36me3), and RNA expression levels, while an opposed trend was observed when considering marks of silent chromatin (H3K27me3 and H3K9me3). Interestingly, the ORC1 iCLIP signal also correlated with H4K20 methylation, recognized by ORC1^9^, as well as with ORC1 chromatin binding determined by ChIP-seq (Fig. 1h and Supplementary Fig. 4a–f).

To further investigate the relationship between ORC1-RNAs and DNA replication, we mapped the active replication origins in HCT116 cells using Short Nascent Strand sequencing (SNS-seq) (Supplementary Fig. 4g)^30^. SNS-seq identified 37725 origins that were consistent with previously published origin mapping in other cell types^13^, since 63% of the HCT116 origins overlapped with those in quantiles Q1 and Q2 of the 10 defined by the mentioned study, which represent the most robust origins with the highest conservation among cell types^13^ (Supplementary Fig. 4h). Notably, we found the mapped origins to be highly enriched at the TSS of ORC1-RNA genes (*p* value 3.82e^−51^, Supplementary Fig. 4i). More importantly, the level of ORC1 binding to RNA correlated with the SNS-seq signal, directly proportional to origin activation frequency, as shown by the distribution following the iCLIP gene quantiles (Fig. 1i and Supplementary Fig. 4j).

These results indicate the co-occurrence of ORC1 binding to RNA, and the position and activation of origins in *cis*, that is, at the loci where RNAs are transcribed (Fig. 1j).

### RNAs bound by ORC1 are necessary for optimal origin activation

**Fig. 2:**
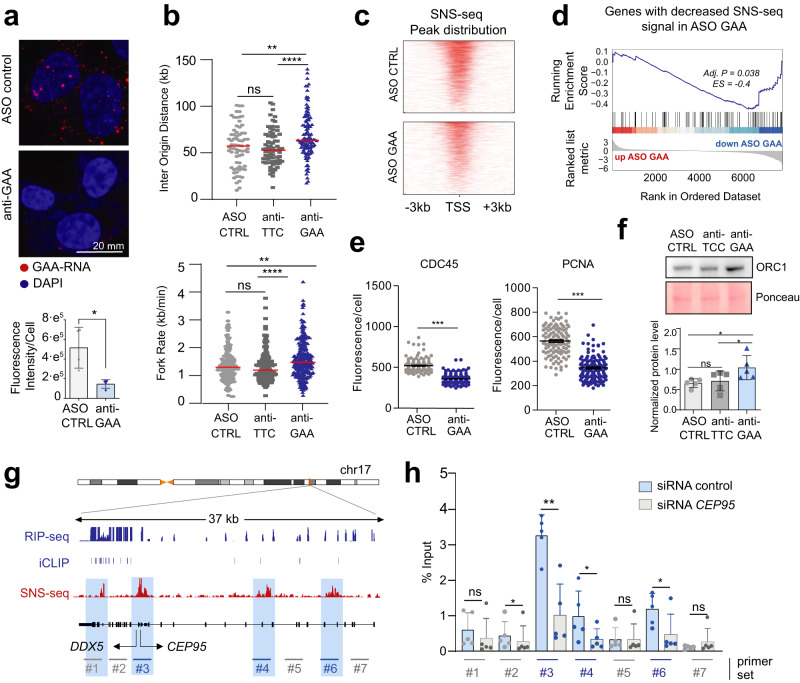
RNAs bound by ORC1 are necessary for optimal origin activation. **a** RNA-FISH representative images, and signal quantification (below), of RNAs containing GAA repeats, in ASO-transfected HCT116 cells. Dots represent mean values (*n* = 4 biologically independent samples) ±SEM. * denotes *p* value < 0.05, derived from unpaired two-tailed Student’s *t*-test. **b** DNA fiber quantification of inter-origin distances and fork rates of ASO-transfected HCT116 cells. Red lines indicate the median. ns denotes *p* value > 0.05, ** denotes *p* value < 0.01, **** denotes *p* value <0.0001, derived from unpaired two-tailed Mann–Whitney *t*-test. **c** SNS-seq peak count frequency and distribution relative to TSS positions (±3 kb) in ASO control and anti-GAA treated HCT116 cells. **d** GSEA showing the reduction of SNS-seq signal (peaks enriched in the control condition) in anti-GAA downregulated genes. Statistical significance (adjusted *p* value 0.038) of the enrichment score (ES) derives from a permutation test. **e** CDC45 and PCNA chromatin immunofluorescences per cell (HCT116) upon ASO knockdown, after soluble protein washout. Data were presented as mean values (*n* > 100 cells) ±SEM. *** denotes *p* value < 0.001, derived from unpaired two-tailed Mann–Whitney *t*-test. **f** ORC1 western blot and protein quantification, in chromatin extracts of ASO-transfected HCT116 cells. Data were presented as mean values (*n* = 5 biologically independent experiments) ±SEM. ns denotes *p* value > 0.05, * denotes *p* value < 0.05, derived from paired two-tailed Student’s *t*-test. **g** Browser snapshot at DDX5-CEP95 locus showing ORC1 RIP-seq enrichment, ORC1 iCLIP peaks, and SNS-seq reads in HCT116 cells, with the position of qPCR primers (#) indicated, and origins highlighted in blue. **h** Enrichment of nascent strands determined by SNS-qPCR at genomic positions indicated in Fig. 2g, in siRNA-treated HCT116 cells. Data were presented as mean values (*n* = 5 biologically independent experiments) ±SEM. ns denotes *p* value > 0.05, * denotes *p* value <0.05, ** denotes *p* value < 0.01, derived from paired two-tailed Student’s *t*-test.

Having determined that ORC1 RNA binding correlates and spatially coincides with origin activation, we addressed whether the RNAs play a role in DNA replication initiation. We first used a global approach by taking advantage of a feature found in many ORC1-RNAs: the presence of several tandem GAA repeats (Supplementary Fig. 3c–e). We designed antisense oligonucleotides with the sequence TTCTTCTTCTTCTTCTTCTTC (ASO anti-GAA) (Supplementary Fig. 5a), which targets RNAs containing tandem GAA repeats for degradation by RNaseH, a strategy previously used to knockdown this type of transcripts^31^. RNA-seq showed downregulation of 73% of the RNAs containing a similar GAA motif (Hypergeometric test, *p* value 1·10^−50^) compared to a scramble control ASO (Supplementary Fig. 5b). The knockdown was independently evaluated by RT-qPCR (Supplementary Fig. 5c), and RNA-FISH, showing a pattern of foci that were strongly reduced with the transfection of the anti-GAA ASO (Fig. 2a).

We then analyzed the effect of GAA-RNAs depletion on DNA replication. To control for effects due to binding of the ASOs to DNA, we also included an ASO targeting the antisense sequence (anti-TTC) (Supplementary Fig. 5a). Interestingly, DNA fiber assays showed that the knockdown of GAA-RNAs caused a defect in the activation of replication origins, since increased inter-origin distances and higher fork velocities were detected compared to controls (Fig. 2b). Indeed, while fewer active origins result in increased distance between origins, the increased fork rate has been described as a mechanism to compensate for origin firing defects^32^. Consistently, sequencing of nascent strands (SNS-seq) showed a decrease of origin activation at TSSs upon knockdown of GAA-RNAs (Fig. 2c), suggesting that the RNA may be important for the normal firing of origins at these genomic sites. Of note, the decreased origin activity preferentially affected genes with downregulated RNAs (GSEA adj. *p* value = 0.038 and Enrichment Score = −0.4, Fig. 2d), pointing to a *cis*-regulatory mechanism of RNA. The detrimental effect on origin activation was also evident when assessing the association to chromatin of the firing factors CDC45 and PCNA, recruiting DNA polymerase to activated origins^33^, which were significantly reduced on chromatin (Fig. 2e and Supplementary Fig. 5d). In contrast, the levels of ORC1 protein on chromatin were increased (Fig. 2f and Supplementary Fig. 5e). These data support that the general inhibition of RNAs bound by ORC1 has a negative effect on origin activation, linked to an increased association of ORC1 to the chromatin. Nevertheless, we cannot formally exclude that this could be partially attributed to proteins encoded by some of the downregulated mRNAs.

To assess the role of RNA in DNA replication initiation with higher specificity and resolution, we analyzed individual gene loci that produce ORC1-RNAs and contain origins of replication. We selected the *CEP95* locus, transcribing *CEP95* mRNA, one of the HC ORC1 RNAs. *CEP95* locus contains an origin at the TSS and two downstream secondary origins, according to the SNS-seq signal in HCT116 cells (Fig. 2g). To specifically address the role of *CEP95* mRNA in origin activation, we depleted it using siRNA, since RNAi can downregulate nuclear RNA without interfering with transcription or chromatin structure^34^ (Supplementary Fig. 6a–c). Then, we quantified origin activity by SNS-qPCR. Interestingly, the depletion of *CEP95* mRNA caused a specific decrease in the activity of origins on and downstream of *CEP95* TSS, not affecting the origin on the upstream neighbor gene *DDX5* (Fig. 2h). The deleterious effect of *CEP95* knockdown on local origin activation was also observed by ChIP of CDC45 and PCNA, showing decreased chromatin enrichments at local origins while distant origins were unaffected (Supplementary Fig. 6d–g). In contrast, *CEP95* overexpression from a plasmid (i.e. in trans) did not cause changes in origin activity (Supplementary Fig. 6d–g), in agreement with a model where transcribing RNAs would be regulating the activity of replication origins found at their site of production. Analogous initiation defects *in cis* were observed when the HC ORC1 RNA *HSP90AA1* was individually depleted (Supplementary Fig. 6a–c). The decrease of local origin activity, visualized in the enrichment of nascent strands, was evident, while no consistent effect was appreciated on distant origins at non-related loci (Supplementary Fig. 6h–j).

### ORC1 RNA binding mutant is impaired in origin activation

**Fig. 3:**
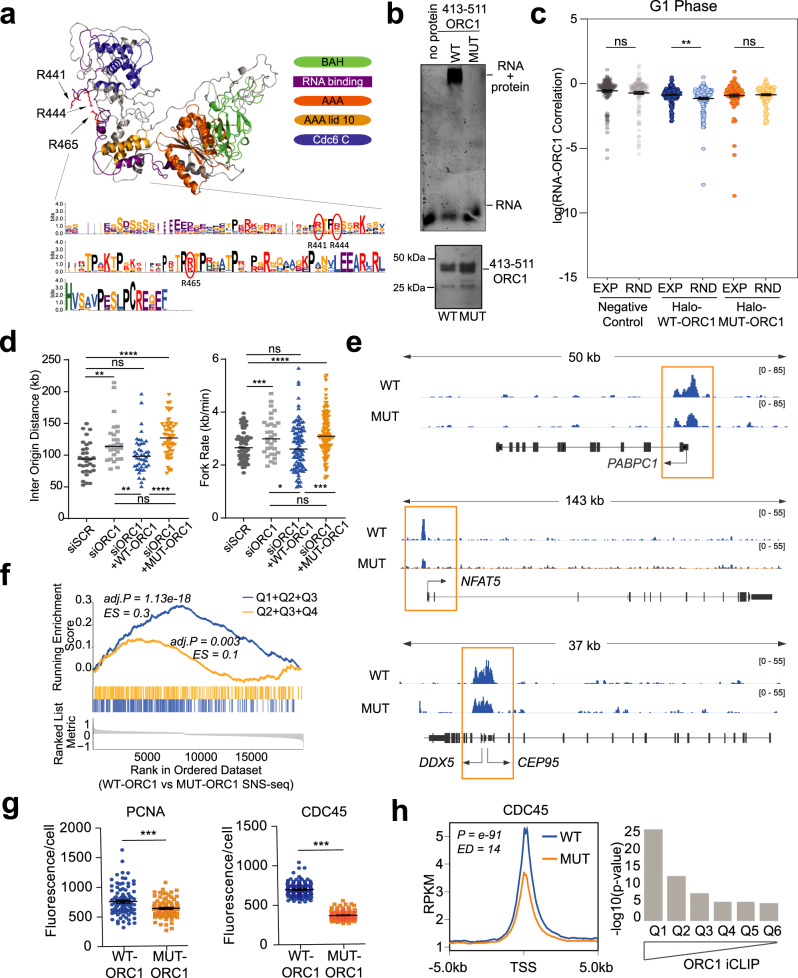
ORC1 RNA-binding mutant is impaired in origin activation. **a** 3D model of human ORC1 showing domains in colors, and residues R441, R444, and R465 (involved in RNA-binding) in red. Below, the vertebrate consensus of ORC1 RNA-binding region, circles indicating mutated residues in MUT-ORC1. **b** RNA staining of EMSA assays, with GST-tagged purified WT and MUT-ORC1 (amino acids 413–511) (2.5 µM) incubated with fragmented cellular RNA (2.5 µM). Below, the silver staining of proteins used in the assay. **c** Cross-correlation between ORC1 and EU-labeled RNA (long pulse) in G1-synchronized U2OS cells, untransfected or transfected with Halo-tagged WT and MUT-ORC1, comparing STORM experimental (EXP) and randomized (RND) samples. Data were presented as mean values (*n* > 50 cells) ± SEM. ns denotes *p* value >0.05, ** denotes *p* value <0.01, derived from unpaired two-sample *t*-test. **d** DNA fiber quantification of inter-origin distances and fork rates in HCT116 cells transfected with the indicated siRNAs, ±plasmids expressing Flag-tagged WT or MUT-ORC1. Black lines indicate the median. ns denotes *p* value >0.05, * denotes *p* value <0.05, ** denotes *p* value <0.01, **** denotes *p* value <0.0001, derived from unpaired two-tailed Mann–Whitney *t*-test. **e** Browser snapshot at ORC1-RNA *PABPC1*, *NFAT5*, and *DDX5-CEP95* loci, showing SNS-seq normalized signal of HCT116 cells stably expressing WT or MUT-ORC1. **f** GSEA showing enrichment of ORC1-RNAs in merged iCLIP-defined quantiles (Q), toward ranked genes according to their WT vs MUT (log2 fold change) SNS-seq coverage at TSSs. Statistical significance (adjusted *p* value 1.13e-18 or 0.003) of the enrichment scores (ES) were calculated by permutation tests. **g** CDC45 and PCNA chromatin immunofluorescences per cell, in HCT116 cells stably expressing WT or MUT-ORC1, after soluble protein washout. Data were presented as mean values (*n* > 100 cells) ± SEM. *** denotes *p* value <0.001, derived from unpaired two-tailed Mann–Whitney *t*-test. **h** Coverage plot of CDC45 ChIP-seq data at TSSs, in WT or MUT-ORC1 HCT116 stably expressing cells, and two-tailed *t*-test statistical results between their coverage at TSSs of ORC1 iCLIP-defined gene quantiles (Q).

To determine how the capacity to bind to RNA is relevant to ORC1 function, we next studied an RNA binding mutant (Fig. 3a). Based on a previous work^24^, and as evidenced by RNA electrophoretic mobility shift assays (Fig. 3b and Supplementary Fig. 7a, b), the substitution to Alanines of three Arginines inside the ORC1 RNA binding region (R441A, R444A and R465A) (Fig. 3a) results in the loss of in vitro RNA binding. The impaired recognition of ORC1-RNAs in cells by the mutant protein was also predicted by *Clever Suite* and *cat*RAPID omics *v2* (Supplementary Fig. 7c, d), while it did not predict affection of ORC1 ability to bind to DNA (Supplementary Fig. 7c). Importantly, the absence of UV-crosslinked RNA in iCLIP experiments (Supplementary Fig. 7e) and the decreased colocalization with RNA by STORM (Fig. 3c) showed experimentally the lack of RNA binding by the mutant ORC1 in cells.

**Fig. 4:**
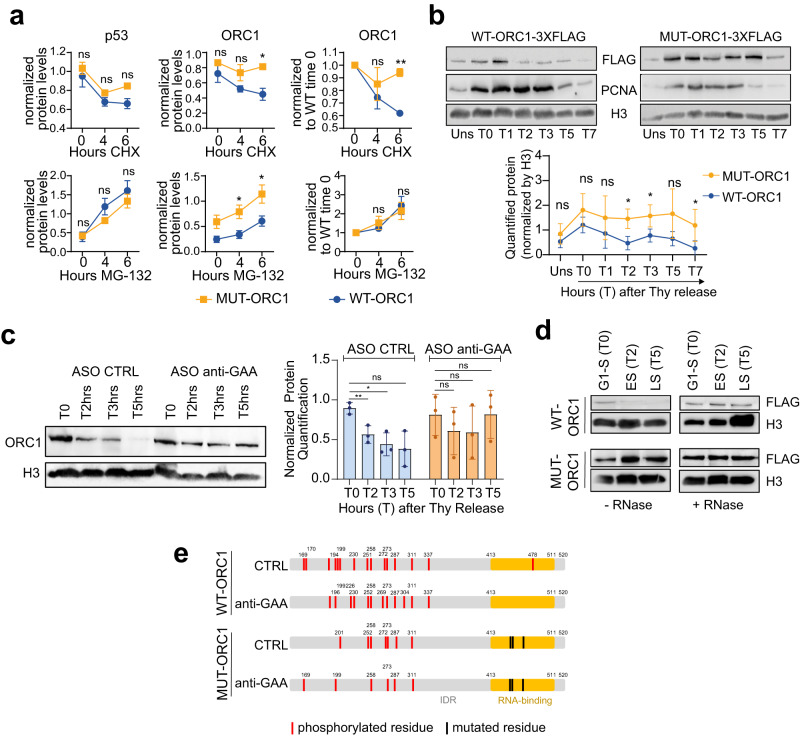
RNA regulates ORC1 chromatin release. **a** p53 and ORC1-3xFlag protein quantification from western blots with total extracts of HCT116 cells, transfected with WT or MUT-ORC1, and treated with cycloheximide (CHX) or MG-132. Dots represent mean values (*n* = 3 biologically independent experiments) ± SEM. ns denotes *p* value >0.05, * denotes *p* value <0.05, ** denotes *p* value <0.01, derived from paired two-tailed Student’s *t*-test. **b** Western blot on chromatin extracts of HCT116 cells, transfected with Flag-tagged WT-ORC1 and MUT-ORC1, unsynchronized (Uns) or synchronized in G1/S and released at different times (T as in Supplementary Fig. 8c). Below, normalized protein quantification. Dots represent mean values (*n* = 4 biologically independent experiments) ± SEM. ns denotes *p* value >0.05, * denotes *p* value <0.05, derived from paired two-tailed Student’s *t*-test. **c** Western blot and quantification of endogenous ORC1 on chromatin in different stages of the cell cycle (T as in Supplementary Fig. 8c), upon depletion of GAA-RNAs (ASO anti-GAA) or control conditions (ASO CTRL). Bars represent mean values (*n* = 3 biologically independent experiments) ± SEM. ns denotes *p* value >0.05, * denotes *p* value <0.05, ** denotes *p* value <0.01, derived from paired two-tailed Student’s *t*-test. **d** Western blot showing the effect of RNase A treatment on WT and MUT-ORC1 chromatin association, along the cell cycle of synchronized cells (T as in Supplementary Fig. 8c). Quantification of independent biological replicates (*n* = 4) is shown in Supplementary Fig. 8d. **e** Representation of the IDR in WT and MUT-ORC1, showing RNA-binding regions (orange), and the discrete positions of RNA-binding mutations (black) and phosphorylated residues (red) detected by mass spectrometry, in control or GAA-knockdown conditions. Source data are provided as a Source Data file.

We next investigated whether the usage of ORC1 RNA binding mutant could affect origin firing. Cells depleted of the endogenous ORC1 and transfected with plasmids expressing WT or MUT-ORC1 were subjected to fiber assays. As expected, depletion of the endogenous ORC1 resulted in the decreased number of fired origins, reflected by increased distances between origins^35^ and compensatory increased fork speed (Fig. 3d and Supplementary Fig. 7f, g). While WT-ORC1 rescued the normal firing and fork rates, MUT-ORC1 did not (Fig. 3d), suggesting that the RNA-binding activity of ORC1 is needed for optimal DNA replication initiation. Notably, SNS-seq showed decreased origin activity in cells stably expressing MUT-ORC1, and preferentially affecting origins at genes that produce the ORC1-RNAs with a stronger level of binding to ORC1 as determined by iCLIP (GSEA Adj. *p* value = 1.13e-18 for genes within Q1-Q3 iCLIP quantiles vs 0.003 for Q4–Q6, and Enrichment scores of 0.3 and 0.1 respectively, Fig. 4e, f and Supplementary Fig. 7h). These results support the idea that ORC1 binding to RNA has a *cis*-regulatory effect on replication origins, as observed upon global (Fig. 2d and Supplementary Fig. 5a–c) or individual (Fig. 2h and Supplementary Fig. 6) RNA knockdown. Also in line with fewer origin activation events observed in GAA-RNA-depleted cells (Fig. 2e and Supplementary Fig. 5d), PCNA and CDC45 were significantly reduced at the chromatin in cells expressing ORC1 RNA-binding mutant (Fig. 3g and Supplementary Fig. 7i), also revealed by ChIP-seq signals, primarily decreased at TSSs of transcribing RNAs bound by ORC1 (Fig. 3h).

We then concluded that the use of ORC1 RNA-binding mutant recapitulates the origin activation defects observed upon knockdown of RNA interactors of ORC1, suggesting that the RNA-binding activity of ORC1 is important for normal initiation of DNA replication.

### RNA binding facilitates ORC1 phosphorylation and chromatin release

To better understand how RNA-binding could be important for ORC1 function, we further investigated the behavior of the RNA-binding mutant by analyzing its chromatin association. Notably, MUT-ORC1 was more associated with the chromatin than its wild-type counterpart (Supplementary Fig. 8a), as was the endogenous ORC1 when ORC1 RNA interactors were depleted (Fig. 2f and Supplementary Fig. 5e). Indeed, MUT-ORC1 presented longer half-life than WT-ORC1, as observed upon translational inhibition with cycloheximide treatment, while both proteins responded to proteasomal inhibition at a similar rate (Fig. 4a), suggesting that the RNA binding activity of ORC1 decreases its stability. Importantly, while the knockdown of GAA-RNAs also led to increased levels of WT-ORC1 on chromatin, it had no effect on the levels of MUT-ORC1 (Supplementary Fig. 8b), indicating that this phenotype is RNA-dependent.

Since origin licensing and firing is governed by the temporal chromatin association and release of ORC along the cell cycle, we decided to dissect this phenotype in synchronized cells. Even though both WT- and MUT-ORC1-expressing cells progressed through S phase at similar rates (Supplementary Fig. 8c), they showed striking differences in ORC1 chromatin association dynamics (Fig. 4b). WT-ORC1, but not MUT-ORC1, was released from the chromatin at the entry of S phase, suggesting that the observed persistence of MUT-ORC1 on chromatin was due to an inefficient protein release. Importantly, endogenous ORC1 also showed delayed chromatin release when GAA-RNAs were depleted (Fig. 4c). Similar effects were observed for WT-ORC1 when synchronized cells were permeabilized and treated with RNase A, resulting in an enhanced chromatin association along the S phase that is progressively lost with no RNase A treatment (Fig. 4d and Supplementary Fig. 8d). In contrast, increased MUT-ORC1 chromatin persistence was observed regardless of RNA degradation (Fig. 4d and Supplementary Fig. 8d). Of note, the delay in MUT-ORC1 release was associated with reduced levels of PCNA loaded on chromatin (Fig. 4b), indicating that the RNA-dependent release of ORC1 could be linked to origin activation.

Given that both origin firing and ORC1 stability are governed by phosphorylation^1^, we hypothesized that the phenotypes observed may account for an RNA-dependent modulation of ORC1 phosphorylation. In line with this hypothesis, MUT-ORC1 showed lower mobility in gels, as did the endogenous ORC1 upon the knockdown of GAA-RNA (Supplementary Fig. 8e). Phosphoproteomic analysis confirmed the hypo-phosphorylation of MUT-ORC1 (Supplementary Data 3), particularly affecting its IDR, where phosphorylated sites are known to regulate ORC1 chromatin release and proteasomal degradation^2^ (Fig. 4e and Supplementary Fig. 8f, g). Decreased phosphorylation of WT-ORC1 was also observed upon GAA-RNA knockdown, while total phosphorylation levels of ORC1 RNA-binding mutant, as well as the number of phosphorylated residues, were less affected by the transient downregulation of ORC1 RNA interactors (Fig. 4e and Supplementary Fig. 8g), indicating that the interaction with RNA plays a role in regulating ORC1 phosphorylation, and subsequent chromatin release and degradation in S phase. Our results suggest a reciprocal regulation of RNA binding and phosphorylation at the IDR of ORC1, which is linked to its chromatin release, and altogether demonstrate that RNA binding dynamically modulates ORC1 function.

## Discussion

Here, through orthogonal approaches, we show that RNA, the product of transcription, is needed for optimal origin activation mediated through the intrinsic RNA binding capacity of ORC1. We demonstrate that RNA transcribed in the proximity of the origins, preferentially found at TSS regions, influences the efficiency at which they are activated. In line with this model, we find an enrichment of ORC1 iCLIP crosslinks at the 5’ region of the RNAs, although not exclusively restricted to it. This is not unexpected given the described mechanism, and the fact that RNA transcripts remain close to the chromatin while co-transcriptionally processed. Moreover, actively transcribed genes are known to form loops, maintaining 3D proximity between the 5’ and 3’ ends^36^. Specifically, we found that the binding of RNA by ORC1 favors the efficient release of the protein at the entry of the S phase. As suggested in previous studies^3^, our data indicates that ORC1 release could be linked to origin firing, although the precise sequence of molecular events at this critical step will require further studies to be fully understood.

Of note, we failed to detect ORC1 RNA binding at a fraction of active origins. While this could be due to limited sensitivity, the RNA-dependent regulation of origins may be restricted to the genes with active transcription, known to replicate in the early S phase, where we find the genes producing ORC1-RNAs. Origins found at transcriptional deserts, associated with late replication, might be activated through a mechanism not involving RNA interaction with ORC1. On the other hand, ORC1 might not be the only sensor of RNA at origins from transcriptionally active regions. RNA may interact with additional proteins in the initiation complex. Among them, CDC6 and CDT1 possess positively charged IDRs^37^ with repetitive sites similar to the observed in ORC1 IDR, although their interaction with RNA has not been studied.

Short linear protein motifs (SLiMs) inside ORC1 IDR have been implicated in ORC1 self-interaction as well as interaction with Protein Phosphatase 1α (PP1α) and CDC6^4^, and, regulated by phosphorylation and dephosphorylation, they contribute to the control of ORC1 levels during the cell cycle. Interestingly, the described SLiMs do not overlap with ORC1 RNA binding domain albeit are part of the same highly flexible region^4^ (Fig. 1a). Thus, the interaction of ORC1 with RNA may influence interactions with these factors and/or other properties linked to the IDR. Among those, ORC1 IDR has been implicated in in vitro liquid-liquid phase separation enhanced by DNA^4,37^, being CDK/Cyclin phosphorylation a key inhibitor of liquid phase condensation by replication initiation factors^37^. While the effect of RNA was not tested in those studies, RNA biophysical properties are consistent with an analogous role. Supporting this notion, we observed that RNA induces the formation of droplets of WT- but not of MUT-ORC1 in a concentration-dependent manner, reaching a threshold where RNA leads to droplet dissolution (Supplementary Fig. 9a–c).

Based on our data, and by analogy to the RNA feedback mechanism described in transcription^38^, where the balance between positive and negative charges determines whether RNA promotes the formation or dissolution of IDR-containing protein condensates, RNA could be playing a sequential role in the initiation of replication (Supplementary Fig. 9d). We speculate that in late M phase, incipient transcription may favor the formation of coacervates that nucleate ORC1 around TSSs, explaining the presence of ORC1 bound to RNAs transcribed from active origins at early-replicating genomic regions, as well as the enrichment of iCLIP binding sites at 5’ ends of genes. Nevertheless, the need of other factors for ORC1 chromatin recruitment should not be excluded. Once preinitiation complexes are assembled on DNA, the property of forming condensates would not be further required. On the contrary, higher local RNA concentration resulting from transcription elongation, particularly at longer genes, would help ORC1 disassembly from chromatin, coupled to an RNA-dependent control of ORC1 phosphorylation balance, and followed by its targeting for degradation and origin firing. This would also explain the enrichment of RNAs containing GAA repeats among ORC1 partners, since these purine-rich sequences are thought to retain RNAs in the nucleus through a saturable nuclear retention factor^29^.

In addition to the described mechanisms, ORC1 is known to have cellular functions beyond the origins of replication^39,40^. It is likely that some of the ORC1-RNA interactions are occurring at those locations. For instance, the interaction between ORC1 and RNA may also be involved in ORC1 transport in and out of the nucleus^41^, which may account for the presence of fully spliced RNAs among ORC1 binders; all questions deserve future investigation.

Overall, our results unveil a novel non-coding function for RNA as a dynamic component of the chromatin, which helps to coordinate transcription and replication in the nucleus.

## Methods

### Cell lines, growth conditions, and culture treatments

HCT116 cells (CCL-247) were cultured in RPMI-1640 (GIBCO), and U2OS cells (HTB-96) in DMEM (GIBCO), all mediums supplemented with 10% fetal bovine serum (GIBCO) and 1x penicillin/streptomycin (Lonza), and maintained at 37 ˚C and 5% CO_2_. To generate HCT116 cells stably expressing WT-ORC1-3xFlag or MUT-ORC1-3xFlag, cells were transfected with pcDNA3.1 vectors containing codon-optimized wild type or mutated cDNA sequences of ORC1 (Supplementary Table 2), and treated with Neomycin-G418 (Sigma) 500 µg/mL for 10 days.

Proteasome inhibition was achieved with short pulses (4 and 6 h) of MG-132 (MilliporeSigma) 50 µM. Cycloheximide (Sigma) was incubated in a culture medium at 100 µg/mL for 4 or 6 h for translation inhibition.

For RNA in vivo labeling, synchronized U2OS cells were incubated with 5-ethynyl uridine (EU- Thermo Fisher) at 0.2 mM final concentration in complete culture medium, in short (20 minutes) or long (16 and 3 h release) pulses.

For fiber assays, exponentially growing HCT116 cells were first pulsed with 50 µM CldU (Sigma) for 20 minutes, washed, and subjected to a second 20 min pulse with 250 µM IdU (Sigma).

In experiments with RNase A treatment, synchronized cells at different points of the S phase (see cellular synchronization section) were trypsinized, permeabilized with 0.05% Tween-20 in PBS for 10 min on ice, and mock-treated or treated with 1 mg/mL RNase A (Sigma) for 30 min at RT, as previously described^42^.

### Cellular transfection

Cellular transfections were performed with Lipofectamine 2000 (Invitrogen) in Serum-free Opti-MEM (GIBCO), following manufacturer instructions.

For RNA knockdown, siRNAs or Antisense Oligonucleotides were transfected for 24 h at final concentrations of 40 or 80 nM, respectively, except for the 48 h transfection to knockdown ORC1. siRNAs were designed using the i-Score designer tool and purchased from Sigma (Supplementary Table 3). Control ASO was designed and synthesized by Ionis Pharmaceuticals. anti-TTC and anti-GAA ASOs were self-designed and synthesized by iDT, with six 2′-o-methoxyethyl nucleotides on the 5′ and 3′ ends, and nine consecutive oligodeoxynucleotides to support RNaseH activity, as previously described^31^ (Supplementary Table 3).

For exogenous ORC1 expression, pcDNA3.1 or pBABE vectors containing codon-optimized wild type or mutated cDNA sequences of ORC1, tagged with 3xFlag or Halo (Supplementary Table 2), were transfected for 48 hours, while, in rescue experiments, plasmid transfection was preceded by 24 h endogenous ORC1 siRNA-mediated depletion (Supplementary Table 3). Exogenous *CEP95* was overexpressed by 48 h transfection of a pcDNA3.1 plasmid purchased from GenScript (Supplementary Table 2).

### Cellular synchronization and cell cycle analysis

For cellular synchronization at G1/S, U2OS cells were subjected to serum starvation for 48 h, while HCT116 cells were blocked by double thymidine shock as previously described in ref. ^43^. Synchronization of HCT116 cells was released by PBS washing and incubation in a complete culture medium, after which cells were harvested at different time points (1, 2, 3, 5, 6, 7, or 9 h) covering the entire S phase.

For cell cycle analysis, cells were fixed in PBS 2% PFA, and incubated in 2 N HCl 0.5% Triton for 30 min. Followed by 0.1 M Na_2_B_4_O_7_ incubation, cells were treated with RNAse A (Promega), resuspended in PBS, and the DNA stained with propidium iodide 1 mg/mL (Sigma). In flow cytometer FACSCalibur, DNA staining was recorded by the BD CellQuest program. Cell cycle profiles were determined by considering the amount of labeled DNA (FL2-H) per cell.

### RNA extraction, processing, and RT-qPCR

Cell preparations were fixed with TRIzol (Sigma), and RNA precipitated with isopropanol.

In bulk RNA-seq experiments of HCT116 cells treated with control or anti-GAA ASOs, total RNA extraction was followed by Turbo DNAse (Invitrogen) digestion and library preparation with TruSeq Stranded mRNA kit from Illumina. Duplicate experiments were sequenced with Illumina NextSeq 500.

For RT-qPCR, up to 1 µg RNA was treated with DNase I (Invitrogen) and reverse-transcribed using the High-Capacity cDNA Reverse Transcription Kit (Applied Biosystem) with random hexamer primers, following manufacturer instructions. The obtained cDNA was analyzed by quantitative PCR (qPCR) using iTaq Universal SYBR Green supermix (Bio-Rad) in a ViiA™ 7 Real-Time PCR System machine (Thermo Fisher), all reactions performed in quadruplicate. *HPRT1* or *GAPDH* RNA levels were used for the normalization of total and cytoplasmic cellular extracts, while *MALAT1* was used to normalize RNA levels in nuclear extracts. For RIP and UV-RIP validations, RNA levels were normalized by their levels in a 10% experimental input of nuclear RNA. Statistical differences between relative RNA levels or relative enrichments were calculated by unpaired two-tailed Student’s *t*-test. RT-qPCR primers were self-designed or designed with the Universal Probe Library Assay Design Center (Roche), and purchased from Metabion (Supplementary Data 4).

### Protein extraction and western blot

Soluble-chromatin cellular fractionation was performed as previously described in ref. ^44^, while subcellular fractionation in cytoplasm and nucleus, and nucleoplasm if indicated, was performed as described elsewhere^45^.

Proteins from cell preparations were quantified with Pierce BCA Protein Assay Kit (Thermo Fisher). Samples were run in denaturing polyacrylamide gels by electrophoresis, and then transferred to nitrocellulose membranes (Bio-Rad). Membranes were blocked with 5% milk in PBS-Tween, and incubated overnight with primary antibodies (Supplementary Table 4). HRP-conjugated secondary antibodies (Cell Signaling Technology, dilution 1:10,000) on the membrane were detected with the use of enhanced chemiluminescence (ECL) reagent (PerkinElmer) in Odyssey CLx (LI-COR), and recorded with Image Studio Lite software. Full scan blots are provided in the Source Data file. Relative protein levels were obtained based on the intensity of the western blot bands using Fiji software. Quantified intensities were normalized to those of the loading reference and, if indicated, fold changes relative to other conditions were calculated. Statistical differences between normalized intensity values were calculated by paired two-tailed Student’s *t*-test.

### ORC1 RIP-seq on nuclear extracts

Native RNA immunoprecipitation was performed as previously described^46^ with minor modifications. Briefly, 40 × 10^6^ asynchronous HCT116 cells, untreated or transiently expressing WT-ORC1-3xFlag, were harvested and lysed. Nuclear lysates were dounced and sonicated (Bioruptor Diagenode) for ten cycles, pre-cleared with protein A/G Dynabeads, and incubated with 5 µg of control IgG (sc-2025) or antibody of interest (anti-FLAG [M2, F1804, Sigma] or anti-ORC1 78-1-172 [Bruce Stillman laboratory^3^]). Protein A/G Dynabeads were added to sequester the antibody, and washed five times. For protein analysis, 10% of beads and inputs were resuspended in 2X Laemmli sample loading buffer, and run in acrylamide gels for western blot. RNA from beads and inputs was obtained following the RNA extraction protocol, to perform RT-qPCR (methods section RNA extraction, processing, and RT-qPCR) or library preparation. For sequencing, samples were first treated with Turbo DNAse (Invitrogen), and libraries were prepared with the TruSeq Stranded Total RNA kit from Illumina. Sequencing of triplicate (anti-ORC1) or duplicate (anti-Flag) experiments was done with Illumina NextSeq 500.

### ORC1 IP and phosphoproteomics

Transfected WT-ORC1-3xFlag and MUT-ORC1-3xFlag proteins in HCT116 cells (Supplementary Table 2), in control or anti-GAA-knockdown conditions (Supplementary Table 3), were immunoprecipitated from nuclear extracts of 160 × 10^6^ cells, as previously described in ref. ^3^, with minor modifications. In brief, after cellular lysis (260 mM Sucrose, 8 mM Tris-HCl pH 7.4, 4 mM MgCl_2_, 0.8% Triton X-100), nuclei were resuspended and incubated for 30 min in high salt buffer (20 mM Tris-HCl pH 7.5, 400 mM NaCl, 0.4% Igepal, 5 mM MgCl_2_, 0.1 mM EDTA, 1 mM CaCl_2_, 10% Glycerol, and 0.1 mM DTT), supplemented with phosphatase and protease inhibitors 1x (Roche), as well as benzonase to digest DNA. After sonication (Bioruptor Diagenode) for 30 cycles, NaCl and Igepal concentrations were brought down to 200 mM and 0.2%, respectively, by adding an equal volume of dilution buffer. Clear nuclear lysates were then pre-cleared with protein G Dynabeads, and incubated with 5 µg of anti-FLAG antibody (M2, F1804, Sigma), untransfected cells being the negative control. Protein G Dynabeads were added to sequester the antibody, and washed five times with complete washing buffer (20 mM Tris, 100 mM NaCl, 0.15% Igepal, 5 mM MgCl_2_, 0.1 mM EDTA, and 10% Glycerol). Beads and inputs were resuspended in 2X Laemmli sample loading buffer, and run in acrylamide gels for Coomassie blue staining.

Bands at the expected molecular weight were cut, and containing proteins were subjected to mass spectrometry. The samples were reduced with 1 mM DTT for 30 min at 60 °C and then alkylated with 5 mM iodoacetamide for 15 min in the dark at room temperature. Gel pieces were then subjected to a modified in-gel trypsin digestion procedure^47^. Gel pieces were washed and dehydrated with acetonitrile for 10 min, followed by removal of acetonitrile, and then completely dried in a speed-vac. Rehydration was done with 50 mM ammonium bicarbonate solution containing 12.5 ng/µL modified sequencing-grade trypsin (Promega, Madison, WI) at 4 °C. Samples were then placed in a 37 °C room overnight. Peptides were later extracted by removing the ammonium bicarbonate solution, followed by one wash with a solution containing 50% acetonitrile and 1% formic acid. The extracts were dried in a speed-vac (~1 h). For the analysis, samples were reconstituted in 5–10 µl of HPLC solvent A (2.5% acetonitrile, 0.1% formic acid). A nano-scale reverse-phase HPLC capillary column was created by packing 2.6 µm C18 spherical silica beads into a fused silica capillary (100 µm inner diameter × ~30 cm length) with a flame-drawn tip^48^. After equilibrating the column, each sample was loaded via a Famos autosampler (LC Packings, San Francisco, CA) onto the column. A gradient was formed and peptides were eluted with increasing concentrations of solvent B (97.5% acetonitrile and 0.1% formic acid). As each peptides were eluted, they were subjected to electrospray ionization and then they entered into an LTQ Orbitrap Velos Pro ion-trap mass spectrometer (Thermo Fisher Scientific, San Jose, CA). Eluting peptides were detected, isolated, and fragmented to produce a tandem mass spectrum of specific fragment ions for each peptide. Peptide sequences (and hence protein identity) were determined by matching protein or translated nucleotide databases with the acquired fragmentation pattern by the software program, Sequest (ThermoFinnigan, San Jose, CA)^49^. The modification of 79.9663 mass units to serine, threonine, and tyrosine was included in the database searches to determine phosphopeptides. Phosphorylation assignments were determined by the Ascore algorithm^50^. All databases include a reversed version of all the sequences and the data were filtered to between a one and two percent peptide false discovery rate. The position and amount of detected phosphopeptides is presented in Supplementary Data 3. Raw data have been deposited to the ProteomeXchange Consortium via the PRIDE partner repository with the dataset identifier PXD043232.

### ORC1 UV-RIP and iCLIP on nuclear extracts

About 40 × 10^6^ asynchronous HCT116 cells were rinsed in cold PBS and irradiated with 150 mJ/cm^2^ in a Stratalinker 2400 at 254 nm. To isolate cellular nuclei, the first steps of fractionation iCLIP protocol were performed^51^, followed by UV-RIP or iCLIP protocols.

For UV-RIP, fixed nuclear pellets of HCT116 cells transiently expressing Flag-tagged WT-ORC1 were resuspended in RIPA buffer and sonicated (Bioruptor diagenode) for 15 cycles. Solubilized nuclear extracts were pre-cleared with protein G Dynabeads, and 5 µg of control IgG (sc-2025) or anti-FLAG (M2, F1804, Sigma) antibodies incubated overnight at 4 °C. Protein G Dynabeads were then added to sequester the antibody, and washed six times. Immunoprecipitates and inputs were eluted, and Proteinase K (NEB) was incubated for 45 min at 45 °C for protein digestion, followed by RNA extraction and RT-qPCR (see methods section RNA extraction, processing, and RT-qPCR).

iCLIP data of ORC1 was generated by following the iCLIP method described elsewhere^52^, using 10^6^ HCT116 UV-fixed nuclei previously transfected with ORC1-WT-3xFlag (*n* = 5), ORC1-MUT-3xFlag (*n* = 2) or untransfected cells as control (*n* = 1) (Supplementary Table 2). First, nuclei were lysed in 1 mL of lysis buffer (100 mM NaCl, 50 mM Tris-HCl pH 7.4, 1% Igepal CA-630, 0.1% SDS, and 0.5% Na-Deoxycholate) supplemented with protease inhibitors 1x (Roche). Nuclear lysates were sonicated (Bioruptor Diagenode) for ten cycles at low intensity. Afterward, RNase digestion was performed for 3 min at 37 °C with 0.4 U (*n* = 3) or 1 U (*n* = 2) of RNase (Thermo Scientific, EN0602) in the presence of 4U of Turbo DNase (Invitrogen), to avoid DNA contamination. Cleared supernatant was immunoprecipitated overnight at 4 °C with 5 µg of anti-Flag antibody (M2, F1804, Sigma). Protein–RNA complexes were visualized using pre-adenylated, infrared dye-labeled L3 adapter with the following sequence: /5rApp/AG ATCGGAAGAGCGGTTCAGAAAAAAAAAAAA /iAzideN/AAAAAAAAAAAA/3Bio/. Reverse transcription was performed using RNA-dependent retrotranscriptase Superscript IV (Invitrogen) and barcoded primers (XXXXX) containing UMIs (NNNN): /5Phos/ WWW XXXXX NNNN AGATCGGAAGAGCGTCGTGAT /iSp18/ GGATCC /iSp18/ TACTGAACCGC. Purification of cDNAs following reverse transcription and circularization was performed using AMPure XP beads (Beckman-Coulter, USA) and isopropanol. Libraries were sequenced as single-end 100 bp reads on Illumina HiSeq 4000.

### Chromatin immunoprecipitation (ChIP)

About 40 × 10^6^ asynchronous HCT116 cells, stably expressing Flag-tagged wild type and mutated ORC1 (RNA-binding mutant) (Supplementary Table 2), or transfected to knockdown or overexpress *CEP95* mRNA (Supplementary Tables 2,3) and synchronized in S phase (see cellular synchronization section), were fixed for 30 min with 2 mM DSG, and then crosslinked with 1% formaldehyde for 10 min. Pelleted cells were lysed (5 mM Tris-HCl pH 8, 85 mM KCl, and 0.5% Igepal), nuclei resuspended in RIPA buffer, supplemented with protease inhibitors 1x, and sonicated (Bioruptor Diagenode) for 30 cycles. Solubilized nuclear extracts were pre-cleared with protein A/G Dynabeads, and 5 µg of control IgG (2729 Cell Signaling or sc-2025) and anti-CDC45 (11882, Cell Signalling Technology) or anti-PCNA (ab29, Abcam) antibodies incubated overnight at 4 °C. Protein A/G Dynabeads were then added to sequester the antibody, and washed with low salt (0.1% SDS, 1% Triton X-100, 2 mM EDTA, 20 mM Tris-HCl pH 8, 150 mM NaCl), high salt (Low Salt buffer with 500 mM NaCl) and LiCl (0.25 M LiCl, 1% Igepal, 1% Deoxycholate, 1 mM EDTA, 10 mM Tris-HCl pH 8) buffers, supplemented with protease inhibitors 1x (Roche). Immunoprecipitates and inputs were eluted, RNA digested with RNase A (Promega) for 30 minutes at 37 °C, and Proteinase K (NEB) incubated for 45 min at 45 °C for protein digestion. Samples were de-crosslinked overnight at 65 °C, followed by phenol:chloroform DNA extraction and ethanol precipitation.

qPCR of precipitated DNA was done as cDNA samples (see RNA processing section), with self-designed primers at genomic DNA replication origins or control regions (Supplementary Data 4), having SNS-seq data in wild-type HCT116 cells as a reference and purchased from Metabion. ChIP-qPCR enrichment was done compared to a 10% input, and relativized to transfection controls. Statistical differences between relative enrichments were calculated by unpaired two-tailed Student’s *t*-test.

For sequencing of CDC45 ChIP samples, libraries of duplicate experiments were generated as previously described in ref. ^53^, and sequenced with Illumina NextSeq 2000.

### Nascent strand preparation by λ-exonuclease method and RT-qPCR analysis

Nascent strand preparation was performed as previously described^54^, in HCT116 cells, wild type or stably expressing WT-ORC1-3xFlag or MUT-ORC1-3xFlag (Supplementary Table 2), untreated or transfected with control or specific siRNAs or ASOs for RNA knockdowns (Supplementary Table 3). SNS-seq from wild-type HCT116 cells was performed in triplicate experiments. To assess origin activity in HCT116 cells knocked down for GAA-RNAs, duplicate SNS-seq experiments were analyzed in cells transfected with a control ASO, or the anti-GAA ASO (Supplementary Table 3). SNS-seq from HCT116 cells stably expressing WT or MUT-ORC1 were performed in duplicates.

Briefly, genomic DNA of 1–2 × 10^8^ exponentially growing cells was digested with Proteinase K (NEB), precipitated with ethanol, and solubilized in TE buffer pH 8 supplemented with RNase OUT (Invitrogen) for 48 h at 4 °C. Denatured DNA was ultracentrifuged in a seven-step 5 to 20% discontinuous sucrose gradient, with SW40Ti rotor at 24,000 rpm for 20 h at 20 °C. About 1 mL fractions were collected, and the DNA precipitated with ethanol. 10% volume of DNA in each fraction was denatured with 0.2 M NaOH and run onto a 1% alkaline agarose gel (50 mM NaOH and 1 mM EDTA). Followed by neutralization with 1x TAE, Syber Gold (Thermo Fisher) staining was used to visualize the fractionation profile with a UV-Biorad camera. Fractions of interest (0.5–2 kb) were treated twice with PNK (Thermo Fisher) and λ-exonuclease (Thermo Fisher) to remove short-cut DNA and keep newly synthetized DNA, which has a 5’ RNA segment. The efficiency of λ-exonuclease digestion was evaluated by incubation of 10% reaction volume with a digested control plasmid.

qPCR of purified nascent strands was done as cDNA samples (see RNA processing section), with self-designed primers at genomic DNA replication origins or control regions having SNS-seq data in wild-type HCT116 cells as reference (Supplementary Data 4), and purchased from Metabion. SNS-qPCR enrichment was done compared to a 10% input of genomic DNA, and statistical differences of relative enrichments, between control or RNA knocked-down cells, were calculated by paired two-tailed Student’s *t*-test. The associated *p* values were used for heatmap representations.

For nascent strand sequencing (SNS-seq), ssDNA fragments were converted to dsDNA as previously described in ref. ^54^. First, short nascent strands were digested with RNase A/T1 mixture (Thermo Fisher) to eliminate both mRNAs and RNA primers, and facilitate adapters ligation for sequencing. Samples were mixed with random hexamer primer phosphate (Roche), and a reaction with the Klenow exo- polymerase (NEB) was used to extend the primers and synthetize the complementary DNA strand. Taq DNA ligase (NEB) was used to ligate the synthetized fragments, and the dsDNA was extracted and precipitated with ethanol. For library preparation, a protocol primarily designed for High-throughput chromatin immunoprecipitation (HT-ChIP) was followed^53^. Duplicate or triplicate experiments were sequenced with Illumina NextSeq 2000.

### RNA-FISH

Cultured HCT116 cells on coverslips, and transfected with control or anti-GAA ASOs (Supplementary Table 3), were fixed in PBS 3% PFA, and washed in 2x SSC 50% formamide. Meanwhile, anti-GAA FISH probes, which were self-designed and purchased from iDT, were denaturized at 92 °C for 4 min, cooled down, and diluted in hybridization buffer (50% Deionized Formamide, 2x SSC, 10% Dextran Sulfate) to 25 nM final concentration. FISH probes were incubated on the cells overnight at 37 °C in humidity.

/56-FAM/*T*T*C*T*T*C*T*T*C*T*T*C*T*T*C*T*T*C**T*T*C**T*T*C**T*T*C**T*T*C**T*T*C**T*T*C**T*T*C**T*T*C**T*T*C*/36-FAM/ Cells were washed with 2x SCC 50% Formamide at 55 °C, followed by a wash with 2x SCC at 55 °C, and a wash with 2x SCC. Preparations were blocked (PBS 0.5% Tween, 10% Heat-inactivated Goat Serum, 0.5% Blocking Reagent [Roche]), and incubated with α-FAM-POD antibody (Roche). After washing with 4x SSC, preparations were incubated with TSA-Cy3 (PerkinElmer) diluted in Amplification Diluent (PerkinElmer). Secondary antibodies were washed with 4x SSC, followed by a wash with 4x SSC 0.1% Triton, and a last wash with 4x SSC. Cells were mounted on microscope glass slides with the mounting solution with DAPI (Vectashield) and imaged with confocal fluorescence microscope Zeiss LSM 880 NLO 63x objective, and images were captured with the ZEN microscopy software (Zeiss). Fiji software was employed for stacks deconvolution and signal quantifications. Probe fluorescence per cell (15 cells each) in 4 independent biological replicates were compared between control and anti-GAA treated cells by unpaired two-tailed Student’s *t*-test.

### Protein imaging (chromatin immunofluorescence, STORM)

Protein imaging at chromatin of HCT116 cells, after removing soluble cellular fractions, was performed as previously described in ref. ^55^, with anti-CDC45 (Cell Signalling 11881) and anti-PCNA (sc-56) antibodies. After immunofluorescence staining with secondary antibodies, coverslips were mounted on microscope glass slides with a mounting solution with DAPI (Vectashield). Preparations were imaged using the 63x objective of the automated microscope Zeiss Axio Imager M1, and images were captured with the ZEN microscopy software (Zeiss). Fiji software was employed for stacks deconvolution and signal quantifications. After delimiting cellular nuclei, fluorescence intensities were measured and normalized by nuclei area. Comparisons between cells (>100) in different experimental conditions were done by unpaired two-tailed Mann–Whitney *t*-test. Results were validated in two independent biological replicates.

In STORM experiments, synchronized U2OS cells (see cellular synchronizatio*n* section), untreated, mock-transfected, or transiently expressing Halo-tagged wild type and mutated ORC1 (Supplementary Table 2), were permeabilized before fixation, to capture protein and RNA imaging at chromatin, as previously described in refs. ^55,56^. Incorporated EU (see culture treatments section) was detected with the Click-it Plus Kit (Thermo Fisher) AF647 (Thermo Fisher), and ORC1 with anti-ORC1 antibody (F-10) (sc-398734) or Janelia Fluor® 549 HaloTag® Ligand (Promega, GA1110). After immunofluorescence staining with secondary antibodies, coverslips were mounted on microscope glass slides with freshly prepared super-resolution imaging buffer (PBS 1 mg/mL Glucose Oxidase [Sigma], 0.02 mg/mL Catalase [Sigma], 10% Glucose [Sigma], 100 mM Mercaptoethylamine [Thermo Fisher]) flowed through. All raw images were acquired using a custom-built inverse microscope platform (Applied Scientific Instrumentation). Briefly, 639 nm (UltraLaser, MRL-FN-639-1000) and 561 nm (Coherent, Sapphire 561 LPX − 500) laser lines were adjusted to 1.5 and 0.8 kW/cm^2^, respectively. Fluorescence emission was expanded with a 1.67× achromatic lens tube and was collected on a sCMOS camera (Photometrics, Prime 95B). Fluorescence signals were collected sequentially using the AF647 (Semrock, FF01-676/37) and AF568 (Semrock, FF01-607/36) single-band pass filters in a filter wheel (ThorLabs, FW102C). A 405 nm laser line (UltraLaser, MDL-III-405-500) was introduced to enhance recovery of dark state fluorophores when required. About 2000 Frames at 33 Hz were acquired for each color.

Localization of single molecules and the mapping of the two different channels were carried out in algorithms written in MATLAB as previously described in ref. ^57^. To quantify the degree of colocalization between RNA and ORC1, cross-correlation (Eq. (1)) between the two species was calculated. Similar to the radial distribution function, for two images *Im*_*1*_ and *Im*_*2*_, the correlation magnitude at displacement **r** = (*r, θ*) is defined as

1 $$c\left({{{{{\bf{r}}}}}}\right)=\frac{{\left\langle \delta {\rho }_{1}\left({{{{{\bf{R}}}}}}\right)\delta {\rho }_{2}\left({{{{{\bf{R}}}}}}+{{{{{\bf{r}}}}}}\right)\right\rangle }_{{{{{{\bf{R}}}}}}}}{{\left\langle {\rho }_{1}\left({{{{{\bf{R}}}}}}\right)\right\rangle }_{{{{{{\bf{R}}}}}}}{\left\langle {\rho }_{2}\left({{{{{\bf{R}}}}}}\right)\right\rangle }_{{{{{{\bf{R}}}}}}}}$$

where *ρ*_*i*_ (**R**) denotes the local density of *Im*_*1*_ at location **R** and ${\left\langle {\rho }_{i}\left({{{{{\bf{R}}}}}}\right)\right\rangle }_{{{{{{\bf{R}}}}}}}$ denotes the average density over the entire image, where ${\left\langle \bullet \right\rangle }_{{{{{{\bf{R}}}}}}}$ denotes the average operator over all the location **R**; ${\delta \rho }_{i}\left({{{{{\bf{R}}}}}}\right)={\rho }_{i}\left({{{{{\bf{R}}}}}}\right)-{\left\langle {\rho }_{i}\left({{{{{\bf{R}}}}}}\right)\right\rangle }_{{{{{{\bf{R}}}}}}}$ denotes the fluctuation of the local density at location **R**. As the correlation is not orientation-specific, the 2D $c\left({{{{{\bf{r}}}}}}\right)=c\left(r,\theta \right)$ was further averaged over $\theta$ and plotted as the correlation profile as the function of the radial distance *r*.

In brief, each nucleus was first manually outlined to generate a ROI for independent analysis. EU and ORC1 signals from the same ROI were submitted for cross-correlation analysis to obtain their association magnitudes, whilst the cross-correlation between the two species from different ROIs serve as a control describing random distributions^56^. An unpaired two-sample *t*-test between experimental and randomized data was done to determine the significance of the correlation. The same analyses on cells with no EU incubation were used as the experimental negative control. Results were validated in two independent biological replicates.

### Fiber stretching and staining

500 HCT116 cells transfected with siRNAs (Supplementary Table 3), plasmids to express exogenous ORC1 (Supplementary Table 2), or ASOs (Supplementary Table 3), and pulsed with CldU and IdU (see culture treatments section), were dropped on Superforst Thermo Scientific microscope slides and lysed with spreading buffer (0.5% SDS, 200 mM Tris-HCl pH 7.5, and 50 mM EDTA) in humidity. Slides were tilted at a 10–15° angle to allow the DNA suspension to run slowly down the slide and air dried. DNA fibers were fixed in −20° cold 3:1 methanol:acetic acid and air dried. For fiber staining, DNA was denatured in 2.5 M HCl for 30 min at room temperature, washed with PBS, and blocked (PBS 1% BSA and 0.1% Triton). Slides were then incubated with primary antibodies detecting CldU (ab6326, Abcam) and IdU (347580, BD), in a humidity chamber overnight at 4 °C. After PBS washing, slides were incubated with fluorescent secondary antibodies. After washing, primary anti-ssDNA clone 16–19 (MAB3034) and secondary fluorescent antibodies to label DNA fibers were incubated for 30 min. Preparations were then air dried and mounted with Prolong diamond (Invitrogen). Preparations were imaged using the 40x objective of the automated microscope Zeiss Axio Imager M1, and images were captured with the ZEN microscopy software (Zeiss).

DNA fiber images were analyzed with Fiji software, considering a conversion factor of 1 µm = 2.59 kb. Two parameters were analyzed: fork rate, measuring the length (in kb) of the IdU track and dividing it by the 20 min of the duration of the pulse; inter-origin distance, measuring the distance between adjacent origins (recognized as IdU-CldU-IdU tracks). Comparisons of fork rate and inter-origin distances between experimental conditions were analyzed by unpaired two-tailed Mann–Whitney *t*-test. Results were validated in two independent biological replicates.

### Protein purification

Purified wildtype and mutated (R441A, R444A, and R465A) ORC1 RNA-binding regions (amino acids 413–511), fused to GST, were produced in Shou Waga laboratory^24^. Protein concentrations were estimated by Coomassie blue or silver staining, compared to BSA known concentrations.

Wildtype and mutated full-length MBP-PP-GFP-ORC1-6xHis were expressed and submitted to Ni-NTA beads purification, followed by a second purification with amylose beads, as previously described in ref. ^4^. Briefly, *E.coli* BL21 cells were transformed with fusion plasmids (tagged full-length ORC1) (Supplementary Table 2), grown in LB media at 37 °C until 0.7–0.9 O.D., and induced overnight with 0.3 mM IPTG at 16 °C. Bacterial cells were then pelleted, washed, and lysed with 100 mg/mL lysozyme in buffer A (25 mM Tris pH 7.5, 150 mM NaCl, 0.02% Igepal, 5 mM MgCl_2,_ 5 mM Benzamidine-HCl, 1 mM PMSF, 1x protease inhibitors [Roche], and 10% Glycerol). After centrifugation, the clarified supernatant was incubated with pre-washed Ni-NTA beads for 3 h at 4 °C. Bead-bound proteins were washed with lysis buffer, and eluted with 300 mM imidazole. WT and MUT MBP-PP-GFP-ORC1-6xHis proteins were further purified with amylose beads and eluted with 20 mM maltose. Protein concentrations were estimated by Coomassie blue or silver staining, compared to BSA known concentrations.

### In vitro assays (EMSA, LLPS)

In electrophoretic mobility shift assays (EMSA), wildtype and mutated GST-ORC1 (413–511) (concentrations indicated in figure captions) or control buffer (25 mM HEPES pH 8, 300 mM NaCl, 10% Glycerol, 1 mM DTT), were incubated with RNA (concentration indicated in figure captions) in 20 µL binding buffer (25 mM HEPES pH 8, 10 mM Mg(C_2_H_3_O_2_)_2_, 0.1 mM EDTA, 5% Glycerol), supplemented with BSA 2 mg/mL, 3 mM ATP (NEB), and RNAs in ribonuclease inhibitors (Promega). The binding reaction was incubated at 30 °C for 30 min, and samples were immediately loaded into a pre-run non-denaturing gel for electrophoresis at 4 °C. RNA was stained in Sybr Gold (Thermo Fisher), visualized with a UV-Biorad camera, and quantified with Fiji software. In experiments with total RNA, RNA fragments were obtained by sonicating (Bioruptor diagenode) total extracts of HCT116 cells, and purifying short RNA fragments with miRNA columns (PureLink). Electrophoresis was done in 7.5% polyacrylamide gels. In EMSAs with *CEP95* RNA, RNA fragments were in vitro synthetized with T7 RNA polymerase (Promega), using as a template the PCR products amplified from the pCDNA3-CEP95 vector (Supplementary Table 2 and Supplementary Data 4). Electrophoresis was done in 0.7% agarose gel.

In liquid-liquid phase separation (LLPS) assays, full-length wild type and mutated MBP-PP-GFP-ORC1-6xHis (4 µM) were resuspended in LLPS reaction buffer (50 mM Tris pH 7.5, 100 mM NaCl, 1 mM MgCl2, and 1 mM DTT) supplemented with Prescission protease, in presence or absence of 4 µM of RNA probes previously used (same sequence) in DNA phase separation experiments^4,37^ (GAAGCTAGACTTAGGTGTCATATTGAACCTACTATGCCGAACTAGTTACGAGCTATAAAC), and incubated at 4 °C for 16 h. Following incubation, the reactions were spotted on microscope slides with coverslips and observed immediately with 63x oil immersion objective of the automated microscope Zeiss Axio Imager M1. Images were captured with the ZEN microscopy software (Zeiss). GFP-droplet size was measured with Fiji software.

### CatRAPID analysis

The *cat*RAPID algorithm estimates the binding potential of a protein–RNA pair through van der Waals, hydrogen bonding, and secondary structure propensities, allowing the identification of binding partners with an accuracy of 0.78 or higher^26,58^.

The *cat*RAPID analysis to predict ORC1 direct interactions with ORC1 RIP-RNAs was performed following standard pipelines^59^. Briefly, we used the major RNA isoform for each gene reported in ORC1 RIP-seq experiments, retrieved the ORC1 interaction scores from RNAct^60^, and all transcripts with *p* value >0.01 were filtered out. Two classes were analyzed: depleted RNA, when log2 fold chang*e* <0, and enriched RNA, if log2 fold change >0, and the difference in the predicted *cat*RAPID scores of enriched and depleted was represented (*z*-score) and computed with unpaired two-tailed Student’s *t-*test. We also used the experimental log2 fold chang*e* to rank the two groups of transcripts, enriched and depleted. Equal fractions of enriched (i.e., highest fold changes) and depleted (i.e., lowest fold changes) RNAs were compared. The discriminative power, measured as the Area under the ROC curve (AUC), increases proportionally to the experimental signal and reaches a value of 0.80, indicating strong enrichment of predicted physical interactions.

*cat*RAPID *omics v2* was used to predict RNA interactions of the wild type and mutated ORC1 protein sequences (R441A, R444A, and R465A)^61^. We divided the Interaction Propensity score in bins of width 20 (a.u.). For each of the Interaction Propensity Bins, we calculated the fraction of RNAs that obtained decreased Interaction Propensity score against the mutated ORC1 sequence. We found that the RNAs exhibiting “High Interaction Propensity” scores were enriched targets of WT-ORC1, while those exhibiting ‘Low Interaction Propensity’ scores were enriched targets of MUT-ORC1. This finding reinforces the notion that the identified RNAs by RIP-seq are bona-fide ORC1 direct interactors.

### cleverSuite analysis

To investigate the effect of amino acid mutations on ORC1, we used the *CleverSuite* approach^62^, which uses two protein sequence sets (Positive and Control/Negative) to build a model able to separate them based on physicochemical features (hydrophobicity, secondary structure, charge, etc.). The model can be reused to predict the classification of other sets. We trained 2 models: RNA and DNA binding ability. For the RNA model we used RNA binding proteins^63^ and, as control, a set of proteins that were found in the lysate from the same study. For the DNA model, we used Proteins annotated as “DNA binding” from UniProt and, as control, a sample set of similar size as the DNA binding proteins that were not annotated as “DNA binding” nor “RNA binding”. The two models can be found at the links (http://crg-webservice.s3.amazonaws.com/submissions/2021-09/393018/output/index.html?unlock=8bc230ac56 and http://crg-webservice.s3.amazonaws.com/submissions/2013-12/17868/output/index.html?unlock=f3a7ffa08f). We used the predictive ability of the *CleverSuite* to assess if the ORC1 mutant (R441A, R444A, and R465A) belongs to the positive or control set. Specifically, we tested the hypothesis of whether the ORC1 region, including the mutations, affected its ability to interact with RNA (positive) and/or DNA (control). The results unequivocally showed that mutant ORC1 has decreased RNA binding activity, while its DNA binding activity remains unaffected. In the analysis, we considered different ORC1 fragments centered around the mutations to control the signal-to-noise ratio of *CleverSuite* scores. Specifically, in the RNA-Binding model, all ORC1 WT fragments are predicted as “Positive” (RNA binding) and all the Mutated ORC1 fragments were predicted as “Control”. Importantly, the same regions were predicted as “Positive” for both WT and mutant (DNA binding) when using the second (Control) model. Thus, the DNA binding ability is predicted to be unaffected by RNA-binding mutations.

### Statistical analysis

Experimental data were plotted and analyzed using the GraphPad statistical software, following the statistical analysis for each type of data, specified in each method section and/or figure captions. Most experimental data are represented as the average of at least three biological replicates, indicated at figure captions. Imaging data is presented as a representative experiment with multiple measurements, which was validated in additional biological replicates. The number of replicates in sequencing experiments is specified in each method section.

R software was used for bioinformatic analysis, using the R package ggplot2 to generate different types of plots (https://cran.r-project.org/web/packages/ggplot2/index.html). Significance was obtained using the statistical test corresponding to each type of data analyzed, as explained in each analysis section. In all cases, *p* values were given using the following thresholds: ns for *p* value > 0.05; * for *p* value ≤ 0.05; ** for *p* value ≤ 0.01; *** for *p* value ≤ 0.001.

### RNA-seq, RIP-seq, and ChIP-seq Pipelines

In ASO control and anti-GAA (Supplementary Table 3) RNA-seq of HCT116 cells, and ORC1 and ORC1-3xFlag RIP-seq experiments, QC of sequencing files was performed with FastQC (http://www.bioinformatics.babraham.ac.uk/projects/fastqc/). Fastq files were trimmed with Trimmomatic (v 0.38)^64^, aligned with STAR^65^, reads aligning to GL contigs were removed, and FeatureCounts^66^ was used to quantify the number of reads falling in annotated genes in hg19 human reference genome, downloaded from Ensembl^67^. DESeq2^68^ was used to measure differential expression, being ASO control, and inputs the reference conditions for RNA-seq and RIP-seq analyses, respectively.

RNA-seq public sequences from untreated HCT116 cells^69^ (GSE118051) were trimmed with Trimmomatic (v 0.38)^64^, aligned to the hg38 reference genome, downloaded from GENCODE^70^, using STAR^65^ with parameters: ‘winAnchorMultimapNmax20 -outFilterMultimapNmax 20 -twopassMode Basic’.

Public ChIP-seq sequencing reads from duplicate experiments (1,2) were analyzed using a Nextflow pipeline^71^ with the golden standard of the NF-Core consortia (https://nf-co.re/). From ENCODE^72^: H3K27me3 (ENCFF457PEW), H3K9me3 (ENCFF020CHJ), H3K4me1 (ENCFF531IUP), H3K27ac (ENCFF227RRY), H3K4me3 (ENCFF213WKK), and H3K36me3 (ENCFF059WYR). From Sequence Read Archive (SRA) the following published data^10^: H2A.Z (SRR9850576, SRR9850577), H4K20me1 (SRR9850580, SRR9850581), and H4K20me2 (SRR9850582 and SRR9850583), ORC1 ChIP input (SRR9850586) and IP (SRR9850594 and SRR9850595). Raw sequencing reads were trimmed and quality control was performed to remove poor quality sequences. Adapter-trimmed reads were then aligned to hg38 human reference genome, downloaded from GENCODE^70^, with BWA^73^ under default parameters. Replicates were merged and deduplicated to remove optical reads^74^. To obtain robust estimates of the results, we respected the best practices as suggested by ENCODE consortia^75^. Then, aligned reads passing all ChIP-seq QC metrics were submitted to MACS2 peak calling^76^ comparing no antibody input and corresponding samples using the function “callpeak -g hs -B -q 0.05–fe-cutoff 1.5 –broad”.

Self-generated CDC45-treated ChIP-seq samples were trimmed to remove adapted and low-quality sequenced reads with Trimmomatic (v 0.38)^64^. Bowtie2^77^ was used to align the reads to the hg19 human reference genome downloaded from ENSEMBL^67^. Coverage tracks were generated with bamCoverage^78^, and the average signal at TSS positions (±5 kb) was divided in bins of 10 bp length, with computeMatrix from deeptools, to be analyzed by paired *t*-test (Euclidean distance of 14.0163511182744 and 13.8052506781907 for replicates 1 and 2, respectively; *p* value 3.08e-16 and 7.73e-65 for replicates 1 and 2, respectively). Coverage differences (and associated *p* values) between WT and MUT-ORC1 cells were also measured at TSSs of genes in individual iCLIP-defined quantiles.

### iCLIP analysis

ORC1 iCLIP sequencing reads were analyzed on the iMaps server (Genialis Workspace) using the iCount software^79^ (https://github.com/tomazc/iCount). Briefly, experimental barcodes were removed and sequencing reads aligned with STAR^65^ to hg38 human reference genome downloaded from GENCODE^70^, allowing two mismatches and ten secondary alignments. DNA or chromatin contamination was excluded by aligned data interrogation with infer_experiment.py package from RSeQC software^80^. Unique Molecular Identifiers (UMIs), were used to distinguish and remove PCR duplicates. To determine protein–RNA contact sites, the sequencing read preceding nucleotide was allocated as the crosslink site event.

The presented data refers to analysis from the three experimental replicates of ORC1-3xFlag transfected cells and low (0.4 U) RNase treatment, while non-transfected control was used to corroborate data specificity (Supplementary Table 1). Replicates were merged and a summary of cDNA counts within genes and genic regions were generated with iCount summary function. Assignment of crosslink sites to coding transcripts, non-coding or biotype features, was done by following segmentation hierarchy rules (https://github.com/tomazc/iCount/blob/master/iCount/genomes/segment.py).

For data representation, the iCLIP signal was normalized by sequencing deep and millions of tags (CPM) and binned per nucleotide. Coverage tracks were generated using deepTools^78^, and metagene plots were drawn using normalized coverages between the transcriptional start site (TSS) and the transcriptional termination site (TTS) of genes, defined by the GENCODE^70^ annotation from hg38 human reference genome, using 100 nucleotide bins. Normalized iCLIP data was also plotted in metagenes, in ±10 kb windows around genomic TSS positions.

The significant crosslink signal was normalized by sequencing deep and millions of tags (CPM). Significant contact sites were identified as iCLIP peaks, using the iCount peak function, based on a false discovery rate (FDR) <0.05 comparing specific sites within a window of three nucleotides with randomized data (100 permutations) and within co-transcribed regions (https://github.com/tomazc/iCount/blob/master/iCount/analysis/peaks.py).

To identify RNA motifs mediating ORC1 binding, iCLIP peaks with more than five crosslinks per nucleotide were slop 100 nucleotides both sides and submitted to MEME motif finding algorithm^81^ with parameters “-rna -maxw 6 -maxsize 1000000000 -neg”, comparing positive sites with negative randomized data from SNS-seq experiments and from homologous genomic regions. Although several motif sizes were tested, 6-mers appeared to be the most reliable. G4 predictions were also obtained, by using TetraplexFinder from the QuadBase2 web server (quadbase.igib.res.in)^82^, using pre-set motif configuration (medium stringency G3 L1-7, Greedy search algorithm, Bulge size = 0) and search “+” strand only. Statistical significance was analyzed by two-proportions *z*-test.

ORC1 binding preferences for small nuclear RNAs (snoRNAs) annotated hg38 reference genome, downloaded from GENCODE^70^, were separately and further assessed in experimental snoDB database v.1.2.1 (http://scottgroup.med.usherbrooke.ca/snoDB/ downloaded in December 2020).

### ORC1 interactome analysis (iCLIP-RIP comparison, genomic data, and MEME motifs)

SAMtools^83^ and Bedops^84^ were used to do different types of operations with genomic data, and the UCSC liftOver tool was used to convert coordinates between different genome versions.

Effective ORC1 protein–RNA contact sites from iCLIP were pooled overlapping Ensembl IDs from iCLIP peaks (>5 crosslink sites and <0.05 FDR) and RIP-seq technique (log2 fold change >1 and *p* value < 0.05) based on annotation of hg38 human reference genome, downloaded from GENCODE^70^, defining ORC1 iCLIP-RNAs, RIP-RNAs, and ORC1-RNAs (union iCLIP and RIP) or HC ORC1-RNAs (overlap iCLIP and RIP) (Supplementary Data 2). The hypergeometric test confirmed the significance of the overlap and the union of both techniques, also when applying different iCLIP cut-offs. Furthermore, to study general agreement between ORC1 RIP and iCLIP in identifying the same groups of transcripts, ORC1 bound and not bound groups of transcripts were defined by iCLIP CTPM parting from general transcriptomic data in HCT116 cells^69^. Statistical differences between these two groups of transcripts in terms of CTPM and RIP enrichment (log2 fold change) were analyzed by unpaired two-tailed Student’s *t*-test.

Biotypes and gene length of different groups of genes (ORC1-RNAs, HC ORC1-RNAs, RIP-RNAs, and iCLIP-RNAs) were obtained from Ensembl BioMart^67^, and HCT116 expression data were obtained from Array Express^85^ study E-MTAB-2770 (RNA-seq of 934 human cancer cell lines from the Broad-Novartis Cancer Cell Line Encyclopedia^86^). Negative controls of ORC1-RNAs and HC ORC1-RNAs consisted in an equal number of RNAs with log2 fold change <−0.25 and *p* adj <0.5, or with −0.16 <log2 fold change <0.16 in ORC1 RIP-seq experiment, to consider genes with no ORC1 RNA-binding, with expression in HCT116 cells. Comparisons of gene length and expression between the different groups of genes were statistically interrogated by unpaired two-tailed Student’s *t*-test. The same controls were used to determine whether ORC1-RNAs and HC ORC1-RNAs interact with each other in the 3D nucleus with different frequencies than controls, and Hi-C data of HCT116 cells were obtained from GEO series accession number GSE104333^87^ (untreated synchronized combined MAPQ ≥30). Juicer^88^ tools command dump was used to extract data from the.hic file and obtain KR-normalized intra-chromosomal contact information at 100 kb resolution. Statistical significance in terms of Hi-C contacts between control or genes of interest was analyzed by a two-proportions *z*-test.

To establish ORC1-RNAs localization within early or late replicating regions of the genome, Repli-seq data for HCT116 cells were obtained from ReplicationDomain (https://www2.replicationdomain.com/index.php)^89^ database, hg19 human reference genome (Int90617792 and Int97243322). Significant enrichment of ORC1-RNA and HC ORC1-RNA genes for early-replicating regions was assessed by hypergeometric test, considering the universe of early and late replicating regions genome-wide.

To detect enriched sequence motifs through entire ORC1-bound RNAs, sequences of the largest transcripts were extracted from the list of candidate genes. Input datasets of RNA sequences were run to find significantly enriched motifs on any length with MEME^81^, with respect to a control dataset of the same number of transcript sequences (option -neg) by using the differential enrichment objective function. Negative controls consisted of the same number of randomly selected transcripts from ORC1 RIP-seq data, thus expressed in HCT116 cells.

### SNS-seq analysis

The quality of the sequencing files was assessed with FastQC (http://www.bioinformatics.babraham.ac.uk/projects/fastqc/). Bowtie2^77^ was used to align the reads to the human hg19 reference genome, downloaded from Ensembl^67^, and Picard (http://broadinstitute.github.io/picard) was used to remove duplicate reads.

To compare SNS-seq in control vs anti-GAA cells, statistical analyses were done for each of the experimental replicates, by considering the normalized raw sequencing signal (bamCoverage, RPKM normalization) at TSS (±7.5 kb) genomic positions. The average signal was divided in bins of 10 bp length, with computeMatrix from deeptools, and analyzed by paired *t*-test (Euclidean distances of 1.471843 and 2.139965, for replicates 1 and 2, respectively; Fold change ASO anti-GAA vs ASO control of 0.9872622 and 0.9704698, for replicates 1 and 2, respectively; *p* value 8.85552e-36 and 6.840894e-167 for replicates 1 and 2, respectively).

To compare SNS-seq experiments in WT vs MUT-ORC1 expressing HCT116 cells, we merged raw sequencing signal (bamCoverage, RPKM normalization) at TSS (±5 kb) genomic positions, from the two experimental replicates. The average signal was divided in bins of 10 bp length, with computeMatrix from deeptools, and compared by paired *t*-test (Euclidean distance of 2.96711709492902; Fold change WT vs MUT 1.059799; *p* value 2.2e-16).

Origin peaks were determined following the analysis pipeline in^77^, that uses two peak callers: MACS2^76^ with a threshold of q = 0.1 to identify narrow peaks, and EPIC2^90^ with FDR = 0.1, to detect diffuse peaks. To obtain the common peaks detected with both methods, we used intersectBed from BedTools^91^, with parameters -wa -u (nonreciprocal, report any overlapping features). The final set of peaks considered for subsequent analyses includes those common MACS2 + EPIC2 peaks that are present in at least two out of the three SNS-seq replicates in the wild-type HCT116 samples, and in both replicates anti-GAA and control ASOs.

SNS-seq identified 37725 origins that were consistent with previously published origin mapping in other cell types^13^, since 63% of the HCT116 origins overlapped with those in quantiles Q1 and Q2 of the ten defined by the mentioned study, which represent the most robust origins with the highest conservation among cell types (called core origins)^13^. Significant enrichment of SNS-seq peaks at TSSs of ORC1-RNA genes, also visualized in metagenes of normalized reads, was assessed by a hypergeometric test, considering the presence of SNS-seq peaks at TSS of genes in the entire genome. To compare anti-GAA samples against control samples, peaks were divided into different groups: present only in control replicates, present only in anti-GAA replicates, common peaks (those overlapping anti-GAA and control peaks), and peaks differentially bound in anti-GAA or in control. Differentially bound peaks were determined with DiffBind^92^ (v.2.10.0) and DESeq2^68^ (v.1.22.1) R packages. ChiPseeker^93^ was used for SNS-seq peak annotation, comparison, and visualization in heatmaps around TSS positions (13108 peaks in ASO control; 12198 peaks in ASO anti-GAA).

Differences in SNS-seq (ASO control vs ASO anti-GAA and WT-ORC1 vs MUT-ORC1) in combination with other data (RNA-seq ASO control vs ASO anti-GAA or ORC1 iCLIP) were assessed by Gene Set Enrichment analyses (GSEA, see Combined data and correlation analyses section).

### Combined data and correlation analyses (ORC1 iCLIP, RIP-seq, SNS-seq, RNA-seq)

ChIP-seq, SNS-seq, and RNA-seq data were normalized by RPKM, in non-overlapping bins of 10 nucleotides. Genome-wide tracks of normalized ORC1 iCLIP (crosslinks and iCLIP peaks), SNS-seq, ORC1 RIP-seq, and public ChIP-seq data^10^, were visualized and plotted in the IGV (integrative genomics viewer) browser^94^. Normalized sequencing data was also presented in coverage tracks and metagenes, using deepTools^78^, heatmaps, and violin or bar plots.

To study the correlation between replication origins and chromatin marks at TSSs of genes encoding for ORC1-RNAs, normalized ChIP-seq, SNS-seq, and RNA-seq data were represented in a correlation heatmap, with associated Spearman’s Correlation values.

ORC1 RNA-binding iCLIP data, normalized by sequencing deep and millions of tags and binned per nucleotide (CTPM), was used to define the division of ORC1-RNA genes (union RIP-seq and iCLIP—see Genomic data analysis section) in 6 quantiles (Q1 to Q6), defining levels of direct ORC1-RNA binding. Having determined iCLIP-defined groups of genes, normalized sequencing data was represented and subjected to statistical analyses, to study the correlation between replication origins, chromatin marks, and ORC1 RNA-binding. Statistical differences between quantiles of genes in terms of RNA levels (RNA-seq), SNS-seq, and ChIP-seq, represented in profile, heatmap, violin, or bar plots, were analyzed by unpaired two-tailed Student’s *t*-test.

Gene set enrichment analysis (GSEA) analyses were performed using fgsea^95^ (v.1.22.0) R package with 10,000 permutations to calculate statistical significance. Genes were filtered ranked according to their log2 fold change between anti-GAA vs Control RNA-seq samples, or between the signal across the TSS (±5 Kb) in WT-ORC1 against MUT-ORC1 SNS-seq replicates. In both cases, genes with significant (adjusted *p* value < 0.05) differences between conditions were considered for generating the ranking lists.

Ranked genes according to their upregulation or downregulation in RNA-seq experiments (ASO control vs ASO anti-GAA) were crossed with a list of genes with reduced SNS-seq signal in the anti-GAA condition. This second list of genes was defined by DiffBind^92^ (v.2.10.0), with a cut-off of *p* value < 0.05, to select genes with TSS SNS-seq peaks (see SNS-seq analysis section) enriched in the control condition.

Ranked genes according to their enrichment of SNS-seq signal at TSS (WT vs MUT SNS-seq) were crossed with the list of genes in iCLIP-defined quantiles, individually (Q1, Q2, Q3, Q4, Q5, and Q6) or in combination (Q1 + Q2 + Q3 and Q4 + Q5 + Q6).

### In silico analysis of ORC1 sequence, structure, and conservation

To trace orthologues of human ORC1, sequence datasets for 132 proteomes (Supplementary Data 5) were downloaded from the available databases comprising 53 prokaryotes and 79 eukaryotes.

Homologous sequences of human proteins were identified using Inparanoid^96^, an automatic method that uses pair-wise similarity scores between two proteomes for constructing orthology clusters, calculated using NCBI-Blast. The program was run using default parameters except for the in-paralog confidence cut-off, which we made more stringent (from 0.05 to 0.25). All Inparanoid blasts were run using a threshold *e*-value of 0.01 and different matrices were used in pair-wise comparisons to account for different evolutionary distances: Blossum45 to compare prokaryotes, Blossum62 for eukaryotes, and Blossum80 for comparisons between metazoans.

The L-INS-i model in Mafft^97^ was used to build a multiple sequence alignment (MSA) with the ORC1 orthologous proteins from vertebrates and metazoans (Supplementary Table 5). The alignment was visualized using Jalview^98^ and its quality was manually checked. Consensus sequences logos were generated with WebLogo^99^. We used MEME Suite web platform^100^ to find motifs within the consensus sequence of the vertebrate RNA-binding sequence of ORC1 orthologues and MEME FIMO^101^ to search for the TPR/K motif in the *H. sapiens* genome, Ensembl^67^.

To analyze the domain repertoire of ORC1 orthologues, we ran the Hmmscan program from HMMER 3.2 (hmmer.org)^102^ against the Pfam database (version 32, September 2018)^103^. Non-overlapping hits with scores above the conditional e-value threshold of 0.05 were considered significant.

ORC1 protein secondary structure was predicted with PsiPred^104^, and the MetaDisorder server^105^ was used to predict intrinsic protein disorder using iPDA^106^, PrDOS^107^, Pdisorder (http://www.softberry.com/) and IUPred long^108^.

Phyre2^109^ was used to predict the 3D protein structure of human ORC1 and the model was visualized and colored using the PyMOL Molecular Graphics System (Schrödinger, LLC.).

### Reporting summary

Further information on research design is available in the Nature Portfolio Reporting Summary linked to this article.

## Supplementary information

Supplementary Information

Peer Review File

Description of Additional Supplementary Files

Supplementary Data 1

Supplementary Data 2

Supplementary Data 3

Supplementary Data 4

Supplementary Data 5

Reporting Summary

## Data Availability

The raw data for RIP-seq, iCLIP, RNA-seq, and SNS-seq have been deposited in NCBI’s Gene Expression Omnibus (GEO) and are accessible through GEO Series accession number GSE173452. The mass spectrometry proteomics data have been deposited to the ProteomeXchange Consortium via the PRIDE partner repository with the dataset identifier PXD043232. Raw data for all figures and Supplementary figures is provided in Supplementary Data files, Supplementary Tables, and Source Data file. RNA-seq public sequences from untreated HCT116 cells were obtained from GEO series GSE118051. ChIP-seq public sequences were downloaded from the ENCODE portal (https://www.encodeproject.org/): ENCFF457PEW for H3K27me3, ENCFF020CHJ for H3K9me3, ENCFF531IUP for H3K4me1, ENCFF227RRY for H3K27ac, ENCFF213WKK for H3K4me3, ENCFF059WYR for H3K36me3. Public Repli-seq data for HCT116 cells were obtained from ReplicationDomain (https://www2.replicationdomain.com/index.php) database, Homo sapiens build hg19, files Int90617792 and Int97243322. snoRNAs were confirmed from public dataset snoDB database v.1.2.1 (http://scottgroup.med.usherbrooke.ca/snoDB/). Public HCT116 RNA-seq expression data were obtained from Array Express study E- E-MTAB-2770. Public Hi-C data of HCT116 cells were obtained from GEO series GSE104333 (untreated synchronized combined MAPQ ≥30). Human OCR1 protein post-translational modification sites were obtained with PhosphositePlus (v.6.5.9.3)(https://www.phosphosite.org/). Pfam database (version 32, September 2018) was used for protein domain identification. Source data are provided with this paper.

## Source data

Source Data
