# Supplementary material for: ORC1 binds to cis-transcribed RNAs for efficient activation of replication origins
Source: Nat Commun. 2023 Jul 24;14:4447. doi: 10.1038/s41467-023-40105-3 (PMC10366126; doi:10.1038/s41467-023-40105-3)
Supplement: Supplementary file 1 — Supplementary Information [file 41467_2023_40105_MOESM1_ESM.pdf]

ORC1 binds to *cis*-transcribed RNAs for efficient activation of replication origins

Supplementary information

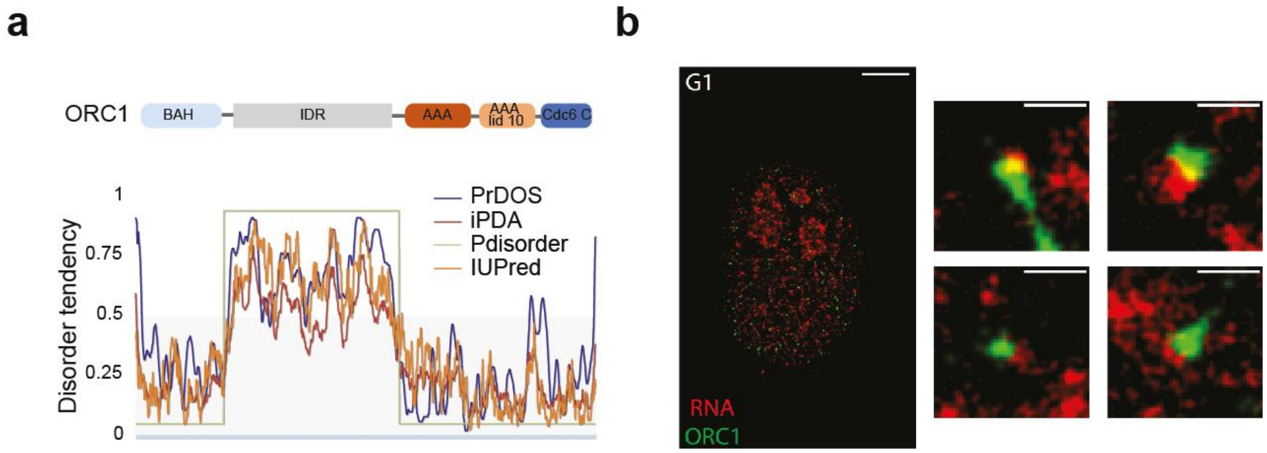

Supplementary Figure 1. ORC1 protein structure and colocalization with RNA in cells.

a, Representation of linearized human ORC1 showing canonical protein domains, as defined by Pfam [1], and the central IDR. Below, disorder plot showing ordered or disordered regions (Disorder Tendency < or > 0.5) along protein residues (x axis), defined by disorder predictors (colored lines). **b,** Representative STORM images of U2OS nucleus labelled with chromatin-associated RNA (long pulse) and ORC1 (Scale bar, 2 µm), and representative zoom-in foci showing their colocalization (quantification of n>50 cells is shown in *Fig. 1b*).

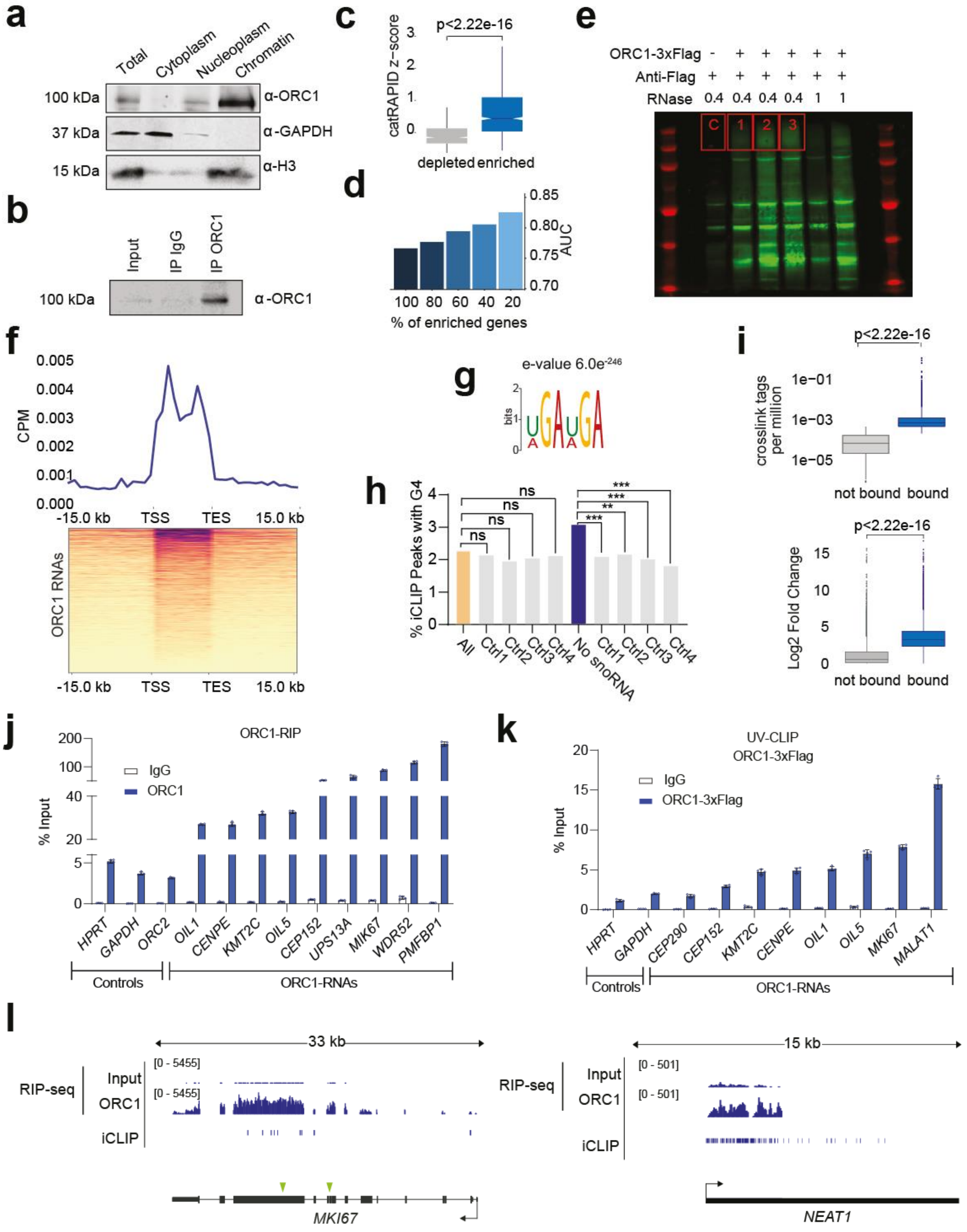

Supplementary Figure 2. Characterization of ORC1 interactome.

a, b, Representative western blots showing (a) ORC1 subcellular distribution, and (b) ORC1 enrichment in RIP protein fraction compared to the experimental input and control IgG (reproduced in biological triplicates). **c,** *cat*RAPID predictions of ORC1 RNA

binding for depleted and enriched RNAs in ORC1 RIP-seq. n= number of genes in each category (*Supplementary Data 1*, ORC1 RIP-seq *p*-value < 0.01), where log2 Fold Change is < 0 for depleted RNAs, or > 0 for enriched RNAs. Box plot shows median distribution between Q1 and Q3. *p*-value derives from unpaired two-tailed Student's *t*-test. **d,** *cat*RAPID predictions of ORC1 physical interactions reporting accurate discrimination (Area Under the ROC Curve, AUC) between enriched and depleted RNAs ranked by RIP-seq fold change. **e,** Immunoprecipitated RNA from anti-Flag iCLIP experiments (representative image of biological triplicates), indicating ORC1-3xFlag transfection and extent of RNase digestion (units), with control (C – untransfected cells) and experimental (1, 2, 3 – ORC1-3xFlag and 0.4 RNase) gel sections analyzed marked in red. **f,** Metagene showing ORC1 iCLIP crosslinks at extended ORC1-RNA entire genes. **g,** MEME motif from RNA windows with ORC1 iCLIP peaks. **h,** G4 content of RNA regions around ORC1 iCLIP peaks compared to control regions, with or without snoRNAs. ns denotes *p*-value > 0.05, ** denotes *p*-value < 0.05, *** denotes *p*-value < 0.01, derived from two-proportions z-test. **i,** ORC1 iCLIP crosslink content (top) and RIP-seq enrichment level (bottom) of transcripts with high or low ORC1 iCLIP-defined binding. n= number of genes in each category (*Supplementary Data 2*, two iCLIP CTPM quantiles). Box plot shows median distribution between Q1 and Q3. *p*-value derives from unpaired two-tailed Student's *t*-test. **j, k,** ORC1-RNA RT-qPCR enrichment in (j) native or (k) UV-crosslinked RIPs (Control IgG, anti-ORC1 or anti-Flag antibodies). n=4 technical replicates from a representative experiment. Bars represent mean values +/- SEM. **l,** Browser capture at *MKI67* or *NEAT1* ORC1-RNAs loci, presenting ORC1 RIP-seq enrichment and iCLIP peaks in HCT116 cells. Green arrows indicate positions of GAA repeats.

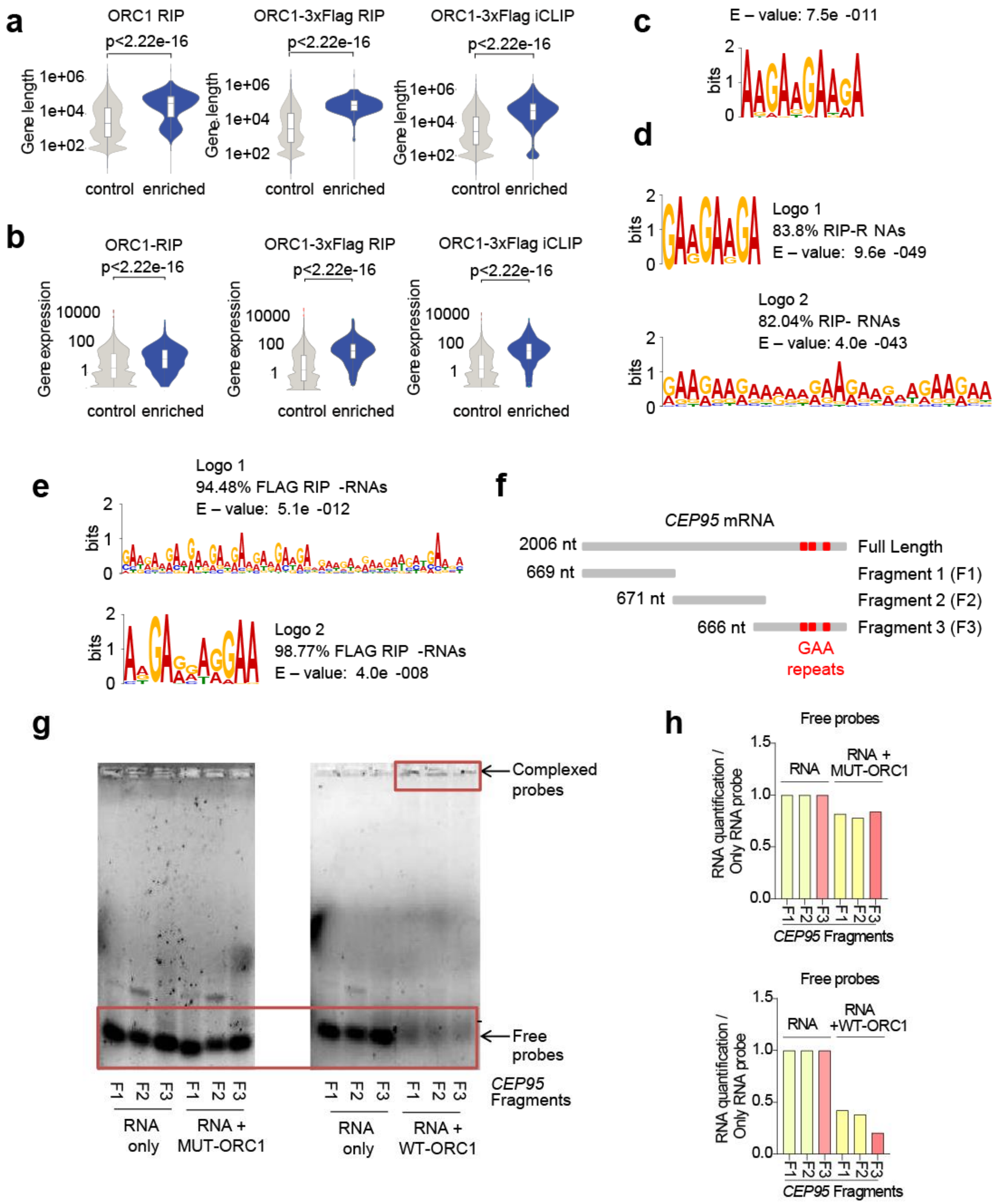

Supplementary Figure 3. Genic features and GAA enrichment in RNAs bound to ORC1.

a, b, Gene (a) length and (b) expression levels of RNAs enriched by either ORC1 RIP-seq, ORC1-Flag RIP-seq, or ORC1-Flag iCLIP, compared to controls. n=number of genes in each category (*Supplementary Data 2;* from iCLIP data [> 5 crosslink sites and

< 0.05 FDR] and RIP-seq data [log2 Fold Change > 1 and p-value < 0.05]). Box plots show median distribution between Q1 and Q3. *p*-values derive from unpaired two-tailed Student's *t*-test. **c, d, e,** MEME motifs from (c) entire sequences of high confidence (HC) ORC1-RNAs, (d) transcripts identified by ORC1 RIP-seq, or (e) ORC1-3xFlag RIP-seq. **f,** Schematic of full length *CEP95* mRNA and *in vitro* synthetized RNA fragments, with GAA repeats shown in red. **g, h,** (g) RNA-stained gels and (h) quantification of EMSA assays with WT and MUT versions of ORC1 RNA-binding domains (amino acids 413-511 – shown in *Fig. 3b*) (2.5 µM), with *CEP95* RNA fragments (2.5 µM) shown in *Supplementary Fig. 3f*.

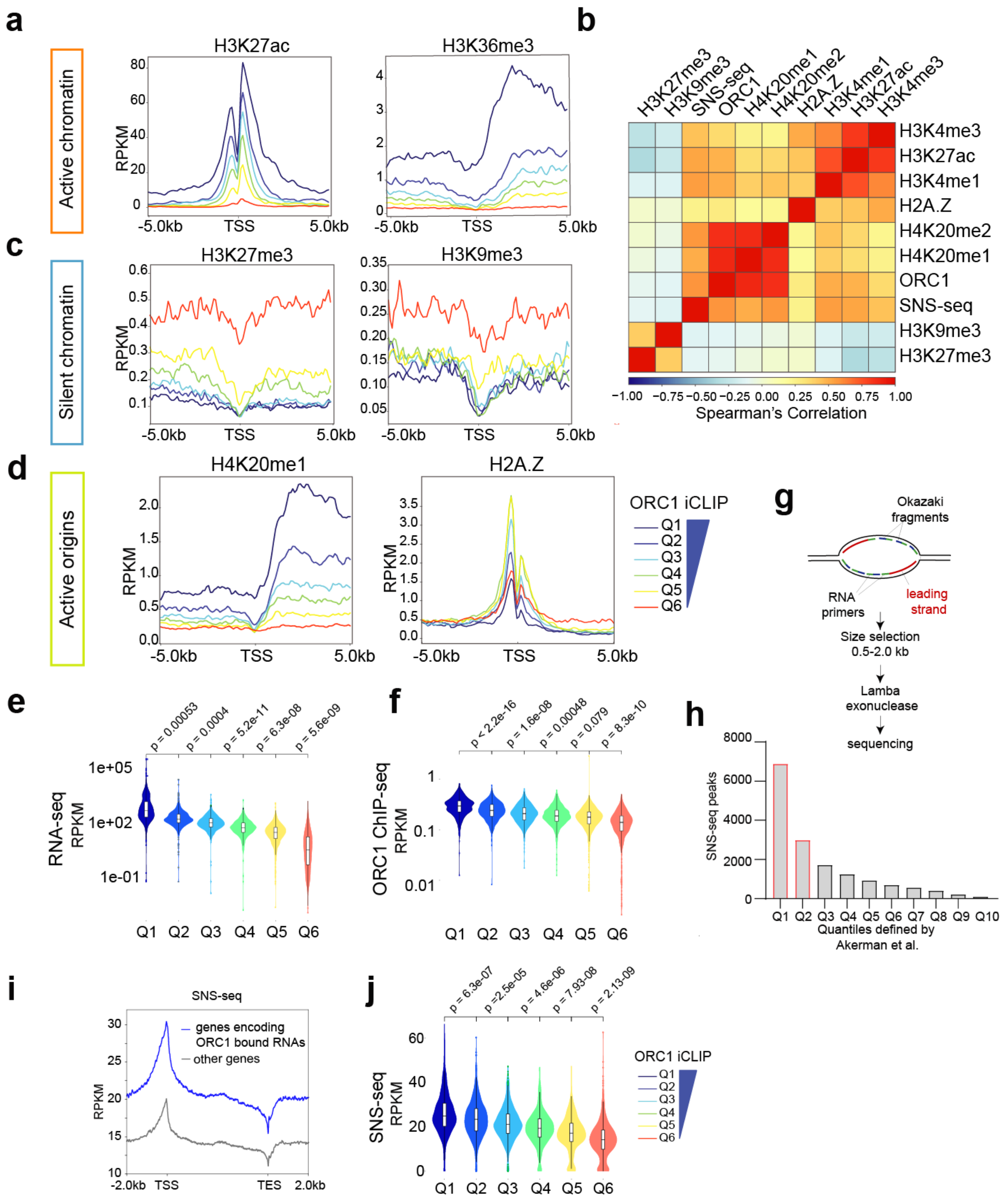

Supplementary Figure 4. SNS-seq detects replication origins at TSSs of transcriptionally active ORC1-RNA genes.

a, Density plots of H3K27ac and H3K36me3 ChIP-seq normalized reads across six ORC1 iCLIP-defined quantiles (Q) of ORC1-RNA genes – color legend shown in *Supplementary Fig. 2d* -, centered around their TSSs (-/+ 5 kb). **b,** Correlation heatmap between ChIP-seq and SNS-seq data around TSSs of ORC1-RNA genes. **c, d,** Density

plots of (c) H3K27me3 and H3K9me3, and (d) H4K20me1 and H2A.Z ChIP-seq data, as shown in *Supplementary fig. 4a* (color legend shown on the right). **e, f,** Normalized (e) RNA-seq and (f) ORC1 ChIP-seq reads at TSSs of iCLIP-defined gene quantiles (Q - color legend shown in *Supplementary Fig. 2d*). n= number of genes in each category (*Supplementary Data 2;* 6 iCLIP derived CTPM quantiles of ORC1-RNA genes). Box plots show median distribution between Q1 and Q3. *p*-values derive from unpaired two-tailed Student's *t*-test. **g,** Schematic representation of SNS-seq protocol. Two replication forks emanate bidirectionally from origins to replicate DNA in RNA-primed leading or lagging strands. Leading strands are selected by size and λ-exonuclease digestion and sequenced. **h,** Number of SNS-seq peaks in untreated HCT116 cells across previously defined SNS peak quantiles (Q) [2]. **i,** Density of normalized SNS-seq reads along bodies of ORC1-RNA or control genes. **j,** Normalized HCT116 SNS-seq reads at TSSs of iCLIP-defined gene quantiles (Q - color legend on the right). n= number of genes in each category (*Supplementary Data 2;* 6 iCLIP derived CTPM quantiles of ORC1-RNA genes). Box plots show median distribution between Q1 and Q3. *p*-values derive from unpaired two-tailed Student's *t*-test.

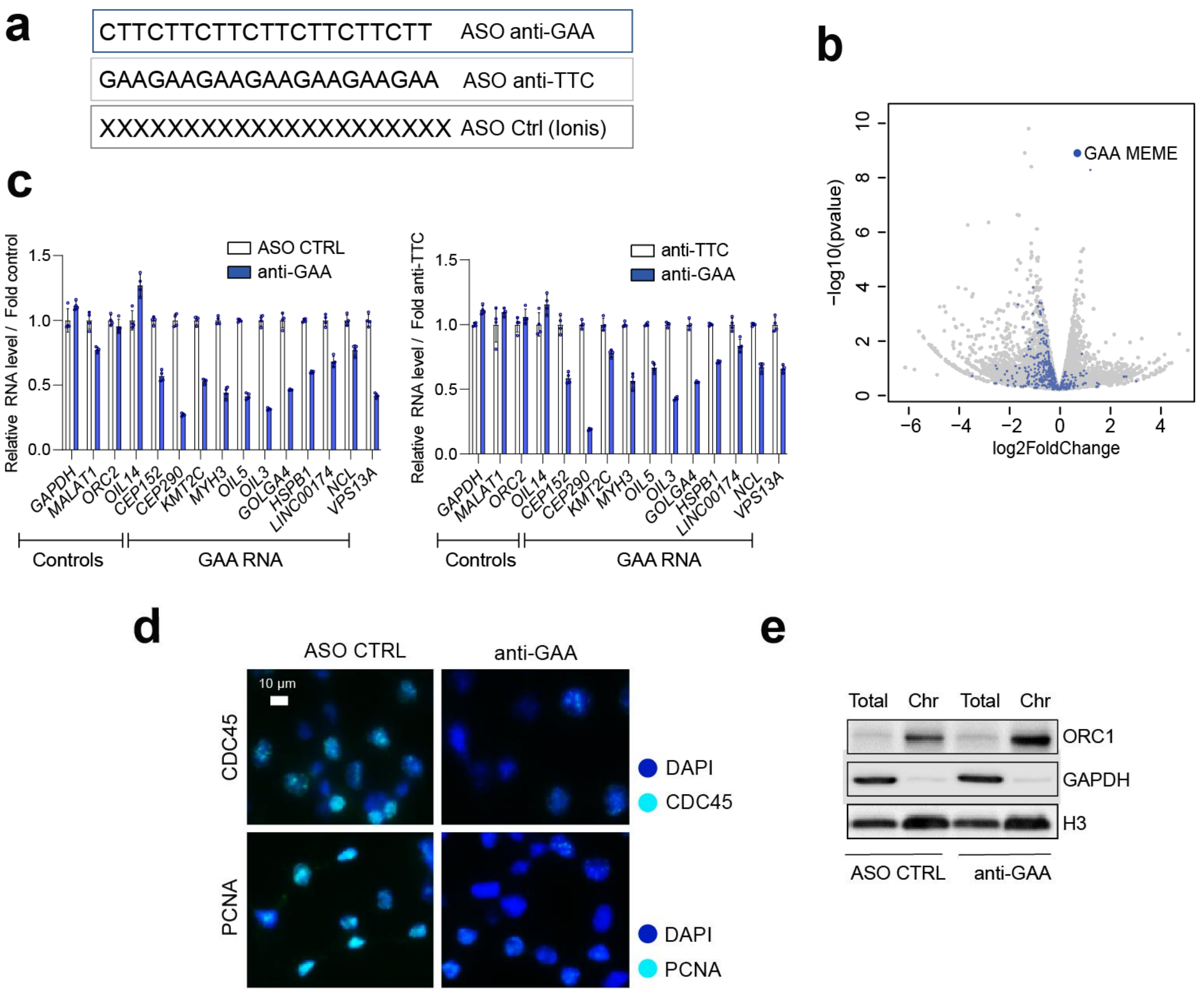

Supplementary Figure 5. ASOs downregulate GAA-RNAs, impacting origin firing. a, Design of ASOs targeting GAA repeats (anti-GAA), the reverse complement sequence (anti-TTC), and a non-targeting control. **b,** Volcano plot of differential expression analysis (RNA-seq) in anti-GAA vs ASO control treated HCT116 cells. RNAs containing GAA sequences bound by ORC1 are shown in blue. **c,** Relative RNA levels of control or ORC1-RNAs containing GAA repeats, in HCT116 cells treated with anti-GAA ASOs, relative to cells treated with control (left) or anti-TTC (right) ASOs. n=4 technical replicates from a representative experiment. Bars represent mean values +/- SEM. **d,** Representative CDC45 and PCNA chromatin immunofluorescence images (reproduced over images of biological triplicates) of HCT116 cells upon ASO treatments, after soluble protein washout. **e,** Representative western blot showing ORC1 in fractioned extracts of ASO-transfected HCT116 cells (reproduced in biological triplicates).

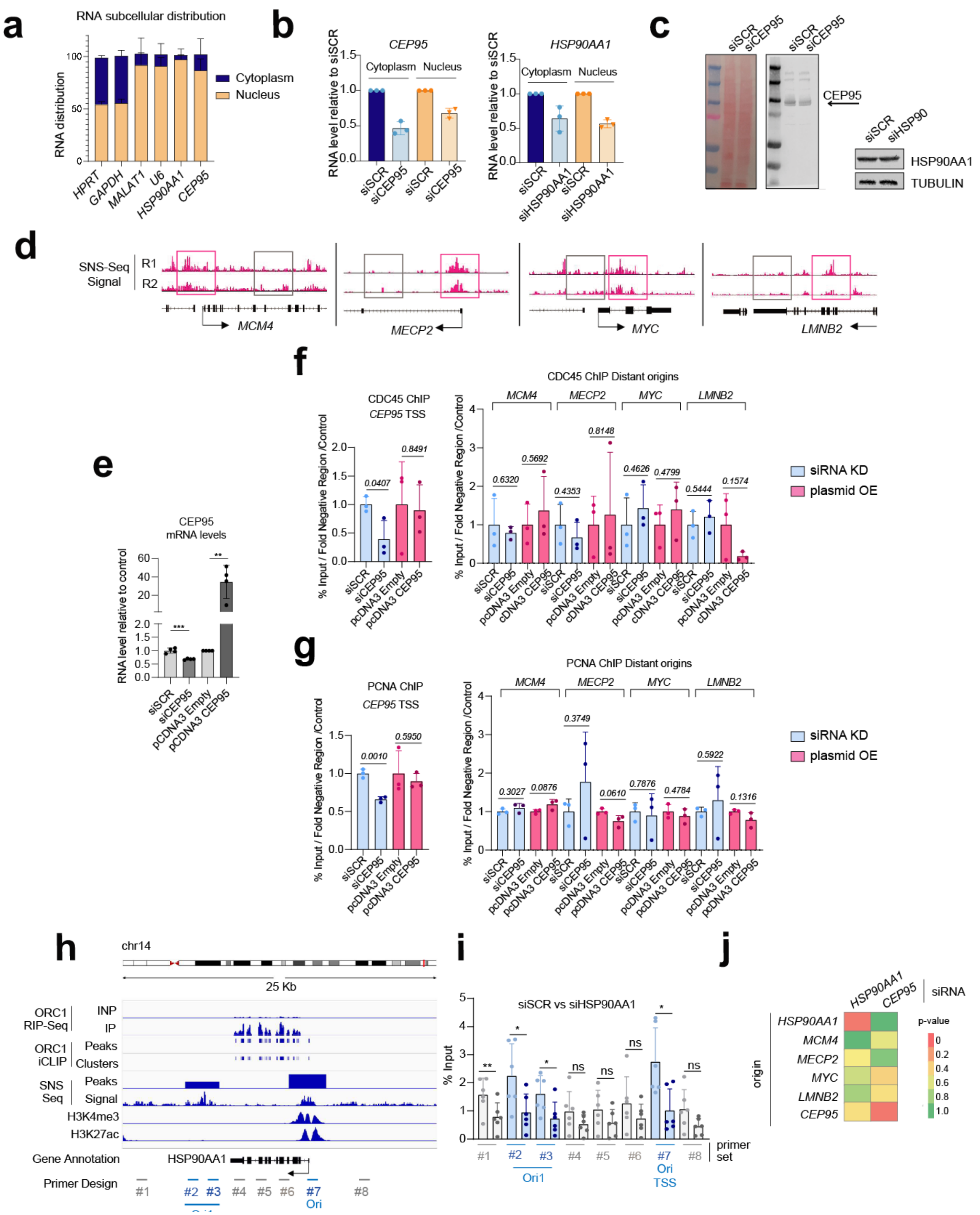

Supplementary Figure 6. Knockdown of ORC1-RNAs reduces origin firing in *cis*. a, Subcellular distribution of RT-qPCR amplified RNAs from nuclear and cytoplasmic cellular fractions of HCT116 cells. Bars represent mean values (n=3 biologically independent experiments) +/- SEM. **b, c,** *CEP95* and *HSP90AA*1 (b) mRNA silencing level (RT-qPCR) in HCT116 cellular fractions after 24 hours siRNAs transfection. Bars

represent mean values (n=3 biologically independent experiments) +/- SEM, (c) not affecting total protein levels. **d,** Browser snapshots of two replicates (R) of SNS-seq data in untreated HCT116 cells, at control genomic positions of *MCM4*, *MECP2*, *MYC*, and *LMNB2* genes. Pink squares indicate SNS-enriched regions compared to proximal control gray squares. **e,** *CEP95* mRNA levels (RT-qPCR) in knockdown (siSCR or siCEP95) and overexpression (pcDNA3 empty or CEP95) conditions, relative to controls. Bars represent mean values (n=3 biologically independent experiments) +/- SEM. ** denotes *p*-value < 0.01, *** denotes *p*-value < 0.001, derived from paired two-tailed Student's *t*-test. **f, g,** Normalized DNA enrichment by (f) CDC45 and (g) PCNA ChIP-qPCR, at local (left) or distant (right) replication origins in conditions presented in *Supplementary Fig. 6e.* Bars represent mean values (n=3 biologically independent experiments) +/- SEM. Indicated *p*-values derive from paired two-tailed Student's *t*-test. **h,** Browser snapshot at *HSP90AA1* locus, showing ORC1 RIP-seq and iCLIP signals, and SNS-seq peaks and normalized reads in untreated HCT116 cells, together with public data of H3K4me3 and H3K27ac ChIP-seq. Paired oligonucleotides (#) target significant replication origins or flanking regions. **i,** qPCR enrichment of nascent strands with primers shown in *Supplementary Fig. 6h*, in siSCR or siHSP90AA1 treated HCT116 cells. Data are presented as mean values (n=6 biologically independent experiments) +/- SEM. ns denotes *p*-value > 0.05, ** denotes *p*-value < 0.05, ** denotes *p*-value < 0.01, derived from paired two-tailed Student's *t*-test. **j,** Heatmap representation of *p-values* evaluating the statistical differences in nascent strand enrichments between siSCR and *CEP95* or *HSP90AA1* knockdowns at the indicated origins (n>5).

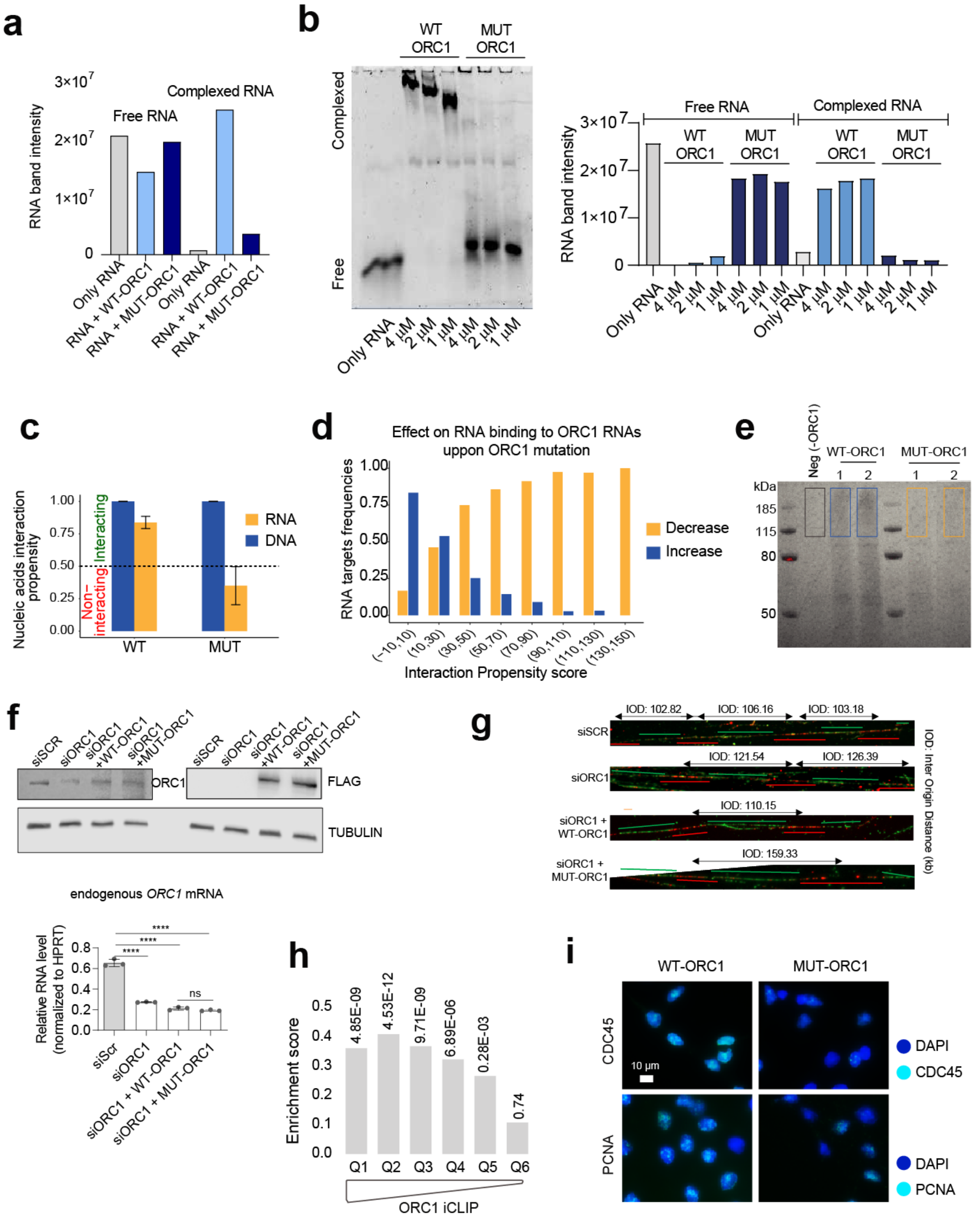

Supplementary Figure 7. MUT-ORC1, deficient in RNA-binding, decreases origin firing.

a, Quantification of stained free or complexed RNA, in EMSA assays shown in *Fig. 3b*.

b, RNA staining and quantification of EMSA assays**,** with increasing concentrations of WT/MUT ORC1 proteins (shown in *Fig. 3b*), incubated with fragmented cellular RNA

from HCT116 cells (2 µM) **c,** *CleverSuite* predicted DNA and RNA binding activity of wild type (WT) and mutant (MUT) ORC1. Data is presented as the mean interaction propensity of 4 different fragments of WT/MUT ORC1 against control samples +/- SD. **d,** *cat*RAPID omics v2 prediction of the increase or decrease of the protein-RNA interaction propensity, caused by the mutation of ORC1 and different bins of ORC1-RNAs. **e,** Staining of immunoprecipitated RNA from control or anti-Flag iCLIP experiments (n=2 biologically independent replicates), in cells transiently expressing WT or MUT-ORC1 tagged with Flag, or untransfected cells as negative control (Neg - ORC1). Boxes indicate gel sections at and above ORC1-expected molecular weight. **f,** Western blots and relative *ORC1* mRNA levels showing knockdown of endogenous ORC1 and overexpression of exogenous WT and MUT-ORC1 tagged with Flag, in rescue experiments shown in *Fig. 3d*. Below, bars represent mean values (n=4 technical replicates from a representative experiment) +/- SEM. ns denotes *p*-value > 0.05, **** denotes *p*-value < 0.0001, derived from unpaired two-tailed Student's *t*-test. **g,** Representative labeled DNA fibers showing distances between interspersed replication origins in WT or MUT-ORC1 rescue experiments shown in *Fig. 3d*. **h,** Enrichment score and associated adjusted *p-values* (permutation test) from GSEA analysis shown in *Fig. 3f* for genes in individual iCLIP-defined gene quantiles. **i,** Representative CDC45 and PCNA chromatin immunofluorescence images in HCT116 cells stably expressing WT or MUT-ORC1 tagged with Flag, after soluble protein washout (reproduced over images of biological triplicates).

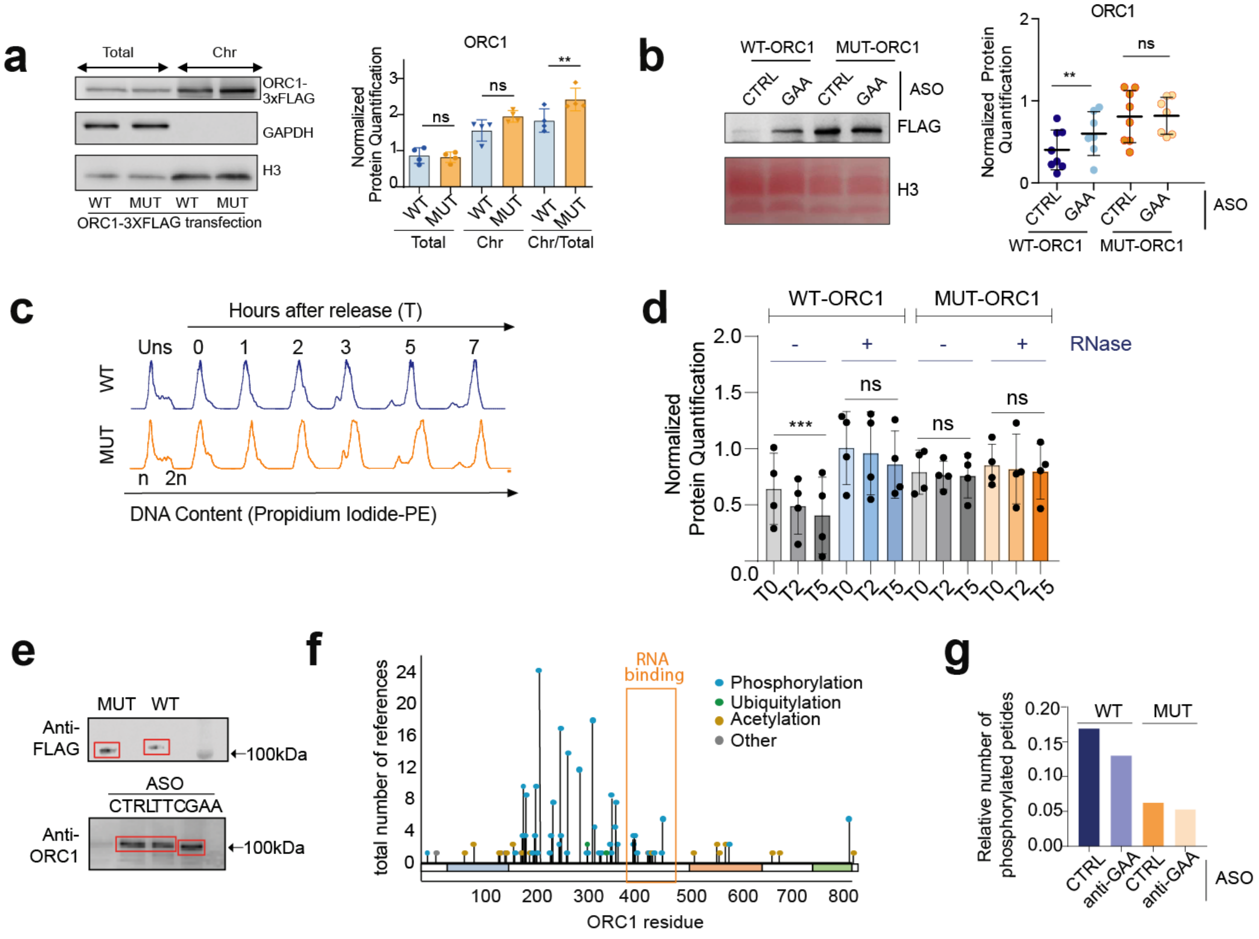

Supplementary Figure 8. RNA-dependent ORC1 phosphorylation controls ORC1 chromatin release.

a, Western blot and protein quantifications of ORC1-3xFlag in total and chromatin extracts of HCT116 cells transiently expressing WT or MUT- ORC1, with calculated chromatin/total ORC1 ratio. Data are presented as mean values (n=4 biologically independent experiments) +/- SEM. ns denotes *p*-value > 0.05, ** denotes *p*-value < 0.01, derived from paired two-tailed Student's *t*-test. **b,** Western blot and protein quantifications of WT or MUT- ORC1-3xFlag in the chromatin fraction of control or GAA-RNA knocked down HCT116 stable cells. Black lines represent mean values (n=8 biologically independent experiments) +/- SEM. ns denotes *p*-value > 0.05, ** denotes *p*-value < 0.01, derived from paired two-tailed Student's *t*-test. **c,** Flow cytometry analysis of synchronized HCT116 cells, transfected with WT or MUT- ORC1-3xFlag, representing DNA content (n or 2n) of unsynchronized cellular populations, or at different hours (T) of thymidine release. **d,** Quantification of western blot presented in *Fig. 4d*, showing WT and MUT-ORC1 chromatin association in synchronized cells (T as in *Supplementary Fig. 8c*) +/- RNase A treatment. Bars represent mean values (n=4

biologically independent experiments) +/- SEM. ns denotes *p*-value > 0.05, ** denotes *p*-value < 0.01, derived from paired two-tailed Student's *t*-test. **e,** Representative western blots showing mobility shift differences between (top) Flag-tagged WT and MUT-ORC1, and (bottom) between endogenous ORC1 in control or GAA-RNA depletion conditions, in HCT116 cells (reproduced in biological triplicates). **f,** PhosphositePlus website representation of referenced ORC1 post-translational modifications along protein residues (x axis), highlighting its RNA-binding region. **g,** Number of detected phosphorylated peptides of WT or MUT-ORC1 by mass spectrometry, relative to the total number of detected peptides, in control or GAA-RNA knockdown conditions.

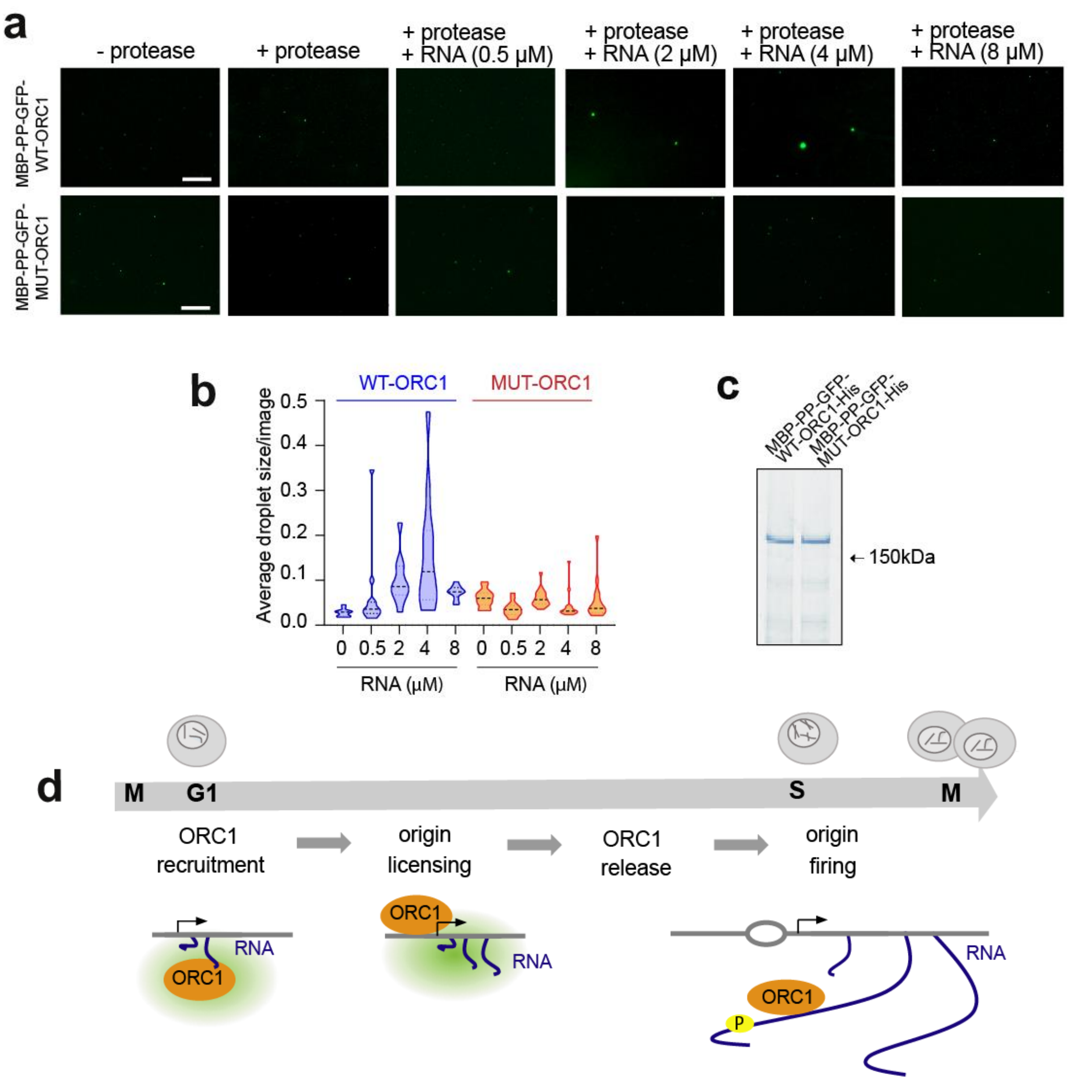

Supplementary Figure 9. Phase separation of ORC1 protein on RNA.
a, Representative images of droplets containing MBP-PP-GFP-WT-ORC1 and MBP-PP-GFP-MUT-ORC1 proteins (shown in *Supplementary Fig. 9c*), treated or untreated with protease to release MBP, and incubated with the indicated concentrations of RNA oligo (Scale bars, 10μm) (reproduced over images of biological replicates). **b,** Quantification of LLPS MBP-PP-GFP-WT-ORC1 or MBP-PP-GFP-MUT-ORC1 droplets as a function of RNA concentrations. Bold dashed lines indicate median; dotted lines indicate quartiles. **c,** Coomassie blue staining of purified full-length tagged WT or MUT-ORC1 (reproduced in biological triplicates). **d,** Speculative model for RNA binding to ORC1 in different phases of the cell cycle. The green area represents liquid-liquid phase separation by ORC1 (and possibly other initiation factors) interacting with RNA.

Supplementary Table 1. iCLIP sequencing reads.

anti-FLAG IP	Uniquely mapped reads	Mapping reads	Multimapping reads	All reads	Unmapped reads
Untransfected (negative control)	764365	2971915	2207550	3583696	611781
ORC1-3xFlag expressing cells (Replicate 1)	7319329	40232831	32913502	41963528	1730697
ORC1-3xFlag expressing cells (Replicate 2)	8333176	44541088	36207912	46127516	1586428
ORC1-3xFlag expressing cells (Replicate 3)	9928271	60729943	50801672	61965266	1235323

Supplementary Table 2. List of plasmids.

Plasmid Number	Backbone	Insert	Comment
1	pCDNA3.1	WT-ORC1-3xFlag	Codon-optimized
2	pCDNA3.1	*MUT-ORC1-3xFlag	Codon-optimized
3	pBABE	Halo-WT-ORC1	Cloned from plasmid 1
4	pBABE	*Halo-MUT-ORC1	Cloned from plasmid 2
5	pCDNA3.1	CEP95	Clone (OHu50782C)
6	pMALp-c2E	MBP-pp-GFP-hORC1wt-6xhis	Stillman Lab
7	pMALp-c2E	*MBP-pp-GFP-hORC1mut-6xhis	

*ORC1 codons for arginines (R), in amino acid positions 441, 444 and 465, were substituted by alanine (A) codon GCC, to generate ORC1 RNA-binding mutant (R441A, R444A, R465A). Plasmids with these cDNA sequences were manually designed and synthetized by GenScript.

Supplementary Table 3. Sequence of siRNAs and ASOs.

Mol.	Name	Sequence
siRNA	si SCR	CAGUCGCGUUUGCGACUGG
siRNA	si *ORC1*	GGUUGUUCCACCGAGAUUCA
siRNA	si1 *HSP09AA1*	CACCAAACATAACGATGAT
siRNA	si2 *HSP09AA1*	TGAAGGAGATGACGACACA
siRNA	si1 *CEP95*	CAGCTTGTCTCACATAACAGGAGAA
siRNA	si2 *CEP95*	CAGAACGCATCAGTGAAACATCTCA
ASO	anti-TTC	/52MOErG/*/i2MOErA/*/i2MOErA/*/i2MOErG/*/i2MOErA/*/i2MOErA/*G*A*A*G*A*A*G*A*A*/i2MOErG/*/i2MOErA/*/i2MOErA/*/i2MOErG/*/i2MOErA/*/32MOErA/
ASO	anti-GAA	/52MOErT/*/i2MOErT/*/i2MOErC/*/i2MOErT/*/i2MOErT/*/i2MOErC/*T*T*C*T*T*C*T*T*C*/i2MOErT/*/i2MOErT/*/i2MOErC/*/i2MOErT/*/i2MOErT/*/32MOErC/

Supplementary Table 4. List of antibodies used for western blotting (1:1,000 dilutions).

Primary Antibodies	Reference
ORC1 (F-10)	sc-398734 (Santa Cruz)
ORC1 (7A7)	sc-23887 (Santa Cruz)
GAPDH (HRP-Conj)	3683 (Cell Signalling)
Tubulin	T5158 (Sigma)
H3	ab1791 (Abcam)
Flag M2	F3165 (Sigma)
PCNA (PC10)	sc-56 (Santa Cruz)
p53	MABE327 (Sigma)
HSP90	4874 (Cell Signalling)
CEP95	MBS3007560 (mYbIOsOURCE)

Supplementary Table 5. ORC1 orthologues. Orthologous proteins from vertebrates and metazoans are listed.

Species	Protein ID	Database
Alligator mississippiensis	A0A151M4M2	Uniprot
Amphimedon queenslandica	A0A1X7VWI9	Uniprot
Anolis carolinensis	G1KHI9	Uniprot
Apis mellifera	A0A088AEY1	Uniprot
Branchiostoma floridae	C3ZFR4	Uniprot
Caenorhabditis elegans	Q9XX17	Uniprot
Callorhinchus milii	V9KDF9	Uniprot
Chelonia mydas	M7B8T8	Uniprot
Crassostrea gigas	K1Q150	Uniprot
Danio rerio	A0A0R4IFH7	Uniprot
Daphnia pulex	E9G6A6	Uniprot
Drosophila melanogaster	O16810	Uniprot
Gallus gallus	Q5ZMC5	Uniprot
Helobdella robusta	T1EDE2	Uniprot
Homo sapiens	Q13415	Uniprot
Ixodes scapularis	B7Q2L8	Uniprot
Latimeria chalumnae	H3AUF8	Uniprot
Lepisosteus oculatus	W5MGU6	Uniprot
Lottia gigantea	V4BTT9	Uniprot
Mnemiopsis leidyi	ML02754a	NHGRI
Monodelphis domestica	F6QFY9	Uniprot
Monosiga brevicollis	A9V9J0	Uniprot
Mus musculus	Q9Z1N2	Uniprot
Nematostella vectensis	A7STK3	Uniprot
Octopus bimaculoides	A0A0L8FWE5	Uniprot
Oikopleura dioica	E4XD69	Uniprot
Ornithorhynchus anatinus	F6TS55	Uniprot
Oryzias latipes	H2MEI0	Uniprot
Oscarella carmela	m.102310	Compagen
Sycon ciliatum	scpid68717	Compagen
Saccoglossus kowalevskii	XP_002738319	NCBI RefSeq
Strigamia maritima	T1JIW5	Uniprot
Trichoplax adhaerens	B3S114	Uniprot
Tribolium castaneum	D6WQP2	Uniprot
Taeniopygia guttata	H0ZF97	Uniprot
Xenopus tropicalis	F6SND7	Uniprot

Supplementary References

1. El-Gebali, S. *et al.* The Pfam protein families database in 2019. *Nucleic Acids Res.* **47**, D427–D432 (2019).
2. Akerman, I. *et al.* A predictable conserved DNA base composition signature defines human core DNA replication origins. *Nat. Commun.* **11**, 4826 (2020).
