# Supplementary material for: ORC1 binds to cis-transcribed RNAs for efficient activation of replication origins
Source: Nat Commun. 2023 Jul 24;14:4447. doi: 10.1038/s41467-023-40105-3 (PMC10366126; doi:10.1038/s41467-023-40105-3)
Supplement: Supplementary file 3 — Description of Additional Supplementary Files [file 41467_2023_40105_MOESM3_ESM.pdf]

Description of Additional Supplementary files

File Name: Supplementary Data 1.
Description: RIP-seq data (ORC1 and ORC1-Flag). RNAs identified by RIP-seq of endogenous and exogenous ORC1 are listed, showing fold changes and *p*-values of replicate experiments.

File Name: Supplementary Data 2.
Description: ORC1 RIP-seq and iCLIP data. (a) ORC1-RNAs are listed, showing gene identification and characteristics, RIP counts and enrichment, and iCLIP crosslink and peak counts. (b) HC-ORC1 RNAs are listed.

File Name: Supplementary Data 3.
Description: Phosphopeptides detected by mass spectrometry. Position of phosphorylated residues in WT and RNA-binding mutant (MUT) ORC1, in control (ASOC) or GAA-RNA (ASO anti-GAA) conditions.

File Name: Supplementary Data 4.
Description: Oligonucleotides. Sequence of oligonucleotides and application.

File Name: Supplementary Data 5.
Description: Sequence datasets used to identify ORC1 human orthologues. 132 proteomes and taxonomy are listed.
