# Supplementary material for: ORC1 binds to cis-transcribed RNAs for efficient activation of replication origins
Source: Nat Commun. 2023 Jul 24;14:4447. doi: 10.1038/s41467-023-40105-3 (PMC10366126; doi:10.1038/s41467-023-40105-3)
Supplement: Supplementary file 9 — Reporting Summary [file 41467_2023_40105_MOESM9_ESM.pdf]

nature portfolio

Corresponding author(s): Maite Huarte

Last updated by author(s): 05/06/2023

Reporting Summary

Nature Portfolio wishes to improve the reproducibility of the work that we publish. This form provides structure for consistency and transparency in reporting. For further information on Nature Portfolio policies, see our Editorial Policies and the Editorial Policy Checklist.

Statistics

For all statistical analyses, confirm that the following items are present in the figure legend, table legend, main text, or Methods section.

n/a	Confirmed	
☐	☒	The exact sample size (*n*) for each experimental group/condition, given as a discrete number and unit of measurement
☐	☒	A statement on whether measurements were taken from distinct samples or whether the same sample was measured repeatedly
☐	☒	The statistical test(s) used AND whether they are one- or two-sided<br>*Only common tests should be described solely by name; describe more complex techniques in the Methods section.*
☒	☐	A description of all covariates tested
☒	☐	A description of any assumptions or corrections, such as tests of normality and adjustment for multiple comparisons
☐	☒	A full description of the statistical parameters including central tendency (e.g. means) or other basic estimates (e.g. regression coefficient) AND variation (e.g. standard deviation) or associated estimates of uncertainty (e.g. confidence intervals)
☐	☒	For null hypothesis testing, the test statistic (e.g. *F*, *t*, *r*) with confidence intervals, effect sizes, degrees of freedom and *P* value noted<br>*Give P values as exact values whenever suitable.*
☒	☐	For Bayesian analysis, information on the choice of priors and Markov chain Monte Carlo settings
☒	☐	For hierarchical and complex designs, identification of the appropriate level for tests and full reporting of outcomes
☒	☐	Estimates of effect sizes (e.g. Cohen's *d*, Pearson's *r*), indicating how they were calculated

Our web collection on statistics for biologists contains articles on many of the points above.

Software and code

Policy information about availability of computer code

Data collection	For generating graphs or curves, GraphPad Prism 8.0.2 software was used.<br>Other plots were produced with R package ggplot2 (https://cran.r-project.org/web/packages/ggplot2/index.html).<br>Image Studio Lite 5.2 software was used to collect western blotting data.<br>QuantStudio Software V1.3 was used to collect quantitative PCR data.
Data analysis	RNA-seq sequences were trimmed with Trimmomatic (v.0.38), aligned with STAR (v.2.6.1), assigned to genes with featureCounts (v.1.6.3) and differentially enriched genes were assessed with DESeq2 en R/Bioconductor.<br>RIP-seq sequences were trimmed with Trimmomatic, aligned with STAR and counted with featureCounts.<br>SNS-seq data were aligned with Bowtie2 (v.2.3.4.2) to align reads, and analyzed with Picard (v.2.18.17), MACS2 (v. 2.1.0.20151222), EPIC2 (v. 0.0.40), deepTools (v.3.2.0) and R/Bioconductor ChIPseeker (v.1.20.0).<br>iCLIP sequencing reads were analyzed on the iMaps server (https://imaps.genias.com/) using the iCount software (https://github.com/tomazc/iCount 18)<br>ChIP-seq sequencing reads were trimmed with Trimmomatic (v.0.38), aligned with Bowtie2 (v.2.3.4.2).<br><br>General genomic data analyses were performed with BedTools (v.2.27.1), SAMtools (v.1.9), Bedops (v2.4.36), deepTools (v.3.2.0) and UCSC liftOver.<br>Juicer tools (v.1.14.08) was used to extract Hi-C data.<br>G-quadruplex prediction was performed with TetraplexFinder from the QuadBase2 web server (quadbase.igib.res.in).<br><br>FastQC (v.0.11.8) and MultiQC (v.1.7) were used for quality control on the sequences.<br>MEME enrichment was assessed by using MEME-ChIP tool (v.5.0.4 and v.5.0.10).

CatRAPID algorithm was used to estimate the binding potential of a protein-RNA pair in order to predict ORC1 direct interactions.
catRAPID omics v2 was used to predict RNA interactions of the wild type and mutated ORC1 protein sequences (R441A, R444A and R465A).
CleverSuite was used to investigate the effect of amino acid mutations on ORC1 in its predicted abilitly to bind RNA and DNA.

ORC1 protein orthology detection was performed with Inparanoid (v.4.1), and sequence alignments and analyses were done using Mafft (v.7.130), Jalview (v.2.8), WebLogo3 and MEME Suite (v.5.1.0). Protein domains were identified using HMMER (v.3.2) Hmmscan.
ORC1 protein secondary structure was analysed with PsiPred (v.4.0) and the MetaDisorder server (iPDA, PrDOS, Pdisorder and IUPred long).
ORC1 protein 3D structure was modeled with Phyre2 and visualised with PyMOL (v.2.4).

GraphPad Prism 8.0.2 software was used for statistical analyses of experimental data.

For manuscripts utilizing custom algorithms or software that are central to the research but not yet described in published literature, software must be made available to editors and reviewers. We strongly encourage code deposition in a community repository (e.g. GitHub). See the Nature Portfolio guidelines for submitting code & software for further information.

Data

Policy information about availability of data

All manuscripts must include a data availability statement. This statement should provide the following information, where applicable:

- Accession codes, unique identifiers, or web links for publicly available datasets
- A description of any restrictions on data availability
- For clinical datasets or third party data, please ensure that the statement adheres to our policy

RNA-seq public sequences from untreated HCT116 cells were obtained from GEO series GSE118051.
ChIP-seq public sequences were downloaded from ENCODE: H3K27me3 (ENCFF457PEW), H3K9me3 (ENCFF020CHJ), H3K4me1 (ENCFF531IUP), H3K27ac (ENCFF227RRY), H3K4me3 (ENCFF213WKK), H3K36me3 (ENCFF059WYR).
Public Repli-seq data for HCT116 cells were obtained from ReplicationDomain (https://www2.replicationdomain.com/index.php) database, Homo sapiens build hg19, files Int90617792 and Int97243322.
snoRNAs were confirmed from public dataset snoDB database v.1.2.1 (http://scottgroup.med.usherbrooke.ca/snoDB/).
Public HCT116 RNA-seq expression data were obtained from Array Express study E-MAT-2770.
Public Hi-C data of HCT116 cells were obtained from GEO series GSE104333 (untreated synchronized combined MAPQ >=30).
Human OCR1 protein post-translational modification sites were obtained with PhosphositePlus (v.6.5.9.3).
Pfam database (version 32, September 2018) was used for protein domain identification.

Own-generated RIP-seq, iCLIP, RNA-seq and SNS-seq have been deposited in Gene Expression Omnibus (GEO) under accession number GSE173452. Protein mass spectrometry raw data has been deposited in Pride, with accession number PXD043232.
Main figures and extended data figures have associated raw data in Supplementary Data, Supplementary Tables, and Source Data files.

Research involving human participants, their data, or biological material

Policy information about studies with human participants or human data. See also policy information about sex, gender (identity/presentation), and sexual orientation and race, ethnicity and racism.

Reporting on sex and gender	N/A
Reporting on race, ethnicity, or other socially relevant groupings	N/A
Population characteristics	N/A
Recruitment	N/A
Ethics oversight	N/A

Note that full information on the approval of the study protocol must also be provided in the manuscript.

Field-specific reporting

Please select the one below that is the best fit for your research. If you are not sure, read the appropriate sections before making your selection.

☒ Life sciences ☐ Behavioural & social sciences ☐ Ecological, evolutionary & environmental sciences

For a reference copy of the document with all sections, see nature.com/documents/nr-reporting-summary-flat.pdf

Life sciences study design

All studies must disclose on these points even when the disclosure is negative.

Sample size	No statistical methods were used to predetermine sample sizes, but determined based on previous experience.

Data exclusions	No data was excluded from the analysis.
Replication	All experiments were reliably reproduced. Each experiment was performed independently at least two times, but usually many more times.
Randomization	All experiments were done with parallelled grown cultured cells.
Blinding	Most of experiments have unambiguous nature of measurements and systematic analyses. Blinding was performed for acquisition and analysis of some experiments requiring imaging (RNA-FISH, DNA fiber immunofluorescence, STORM)

Reporting for specific materials, systems and methods

We require information from authors about some types of materials, experimental systems and methods used in many studies. Here, indicate whether each material, system or method listed is relevant to your study. If you are not sure if a list item applies to your research, read the appropriate section before selecting a response.

Materials & experimental systems

n/a	Involved in the study	
☐	☒	Antibodies
☐	☒	Eukaryotic cell lines
☒	☐	Palaeontology and archaeology
☒	☐	Animals and other organisms
☒	☐	Clinical data
☒	☐	Dual use research of concern
☒	☐	Plants

Methods

n/a	Involved in the study	
☐	☒	ChIP-seq
☐	☒	Flow cytometry
☒	☐	MRI-based neuroimaging

Antibodies

Antibodies used

Western Blot (1:1,000 dilutions):
ORC1 (F-10) sc-398734 (Santa Cruz)
ORC1 (7A7) sc-23887 (Santa Cruz)
GAPDH (HRP Conjugate) 3683 (Cell Signaling)
Tubulin T5158 (Sigma)
H3 ab1791 (Abcam)
Flag M2 F3165 (Sigma)
MCM3 ab97282 (Abcam)
ORC2 (3G6) sc-32734 (Santa Cruz)
ORC4 (H-2) sc-398455 (Santa Cruz)
CDC7 sc-56275 (Santa Cruz)
CDC45 11881 (Cell Signaling)
PCNA (PC10) sc-56 (Santa Cruz)
p53 MABE327 (Sigma)
HSP90 4874 (Cell Signaling)

Western Blot Secondary Antibodies (1:10,000 dilutions)
anti-mouse IgG, HRP-linked Antibody #7076 (Cell Signaling)
anti-rat IgG, HRP-linked Antibody #7077 (Cell Signaling)
anti-rabbit IgG, HRP-linked Antibody #7077 (Cell Signaling)

RIP / UV-RIP: FLAG M2 F1804 (Sigma), ORC1 78-1-172 (Bruce Stillman laboratory), Normal mouse IgG sc-2025 (Santa Cruz).
ChIP: PCNA ab29 (abcam), CDC45 11881 (Cell Signaling), Normal mouse IgG sc-2025 (Santa Cruz), Normal rabbit IgG #2729 (Cell Signaling)
iCLIP: FLAG (M2, F1804, Sigma)
RNA-FISH: α-FAM-POD 11426346910 (Roche), TSA-Cy3 SAT704A001KT (Perkin Elmer)
STORM: AF-647 (ThermoFisher), ORC1 (F-10) sc-398734 (Santa Cruz)
DNA Fiber Immunofluorescence: BrdU antibody [BU1/75 (ICR1)] ab6326 (Abcam), BrdU (B44) 347580 (BD), anti-DNA clone 16-19 (MAB3034)

Validation

All antibodies used are commonly used in the field and have been validated in previous publications/by the manufacturer.
References and manufacturer validations can be found here:
ORC1 (F-10) sc-398734 (Santa Cruz): https://www.scbt.com/p/orc1-antibody-f-10
ORC1 (7A7) sc-23887 (Santa Cruz): https://www.scbt.com/p/orc1-antibody-7a7
GAPDH (HRP Conjugate) 3683 (Cell Signalling): https://www.cellsignal.com/products/antibody-conjugates/gapdh-14c10-rabbit-mab-hrp-conjugate/3683
Tubulin T5158 (Sigma): https://www.sigmaaldrich.com/catalog/product/sigma/t5168?lang=es&region=ES
H3 ab1791 (Abcam): https://www.abcam.com/histone-h3-antibody-nuclear-marker-and-chip-grade-ab1791.html
Flag M2 F3165 (Sigma): https://www.sigmaaldrich.com/catalog/product/sigma/f3165?lang=es&region=ES
MCM3 ab97282 (Abcam): https://www.abcam.com/mcm3-antibody-ab97282.html
ORC2 (3G6) sc-32734 (Santa Cruz): scbt.com/p/orc2-antibody-3g6

ORC4 (H-2) sc-398455 (Santa Cruz): https://www.scbt.com/p/orc4-antibody-h-2
CDC7 sc-56275 (Santa Cruz): https://www.scbt.com/p/cdc7-antibody-spm171
CDC45 11881 (Cell Signaling): https://ocsuat-en.cellsignal.jp/products/primary-antibodies/cdc45-d7g6-rabbit-mab/11881?N=3433506380+4294956287&Nrpp=200&No=%7Boffset%7D&fromPage=plp
PCNA (PC10) sc-56 (Santa Cruz): https://www.scbt.com/p/pcna-antibody-pc10
p53 MABE327 (Sigma): https://www.merckmillipore.com/ES/es/product/Anti-p53-pantropic-Antibody-clone-DO-1,MM_NF-MABE327?ReferrerURL=https%3A%2F%2Fwww.google.com%2F&bd=1
HSP90 4874 (Cell Signalling): https://www.cellsignal.com/products/primary-antibodies/hsp90-antibody/4874
ORC1 78-1-172 (Bruce Stillman laboratory): published in Kara et al. 2015 J. Biol. Chem.
BrdU antibody [BU1/75 (ICR1)] ab6326 (Abcam): published in Terret et al. 2009. Nature
BrdU (B44) 347580 (BD): published in Terret et al. 2009. Nature
anti-DNA (MAB3034): published in Terret et al. 2009. Nature

Eukaryotic cell lines

Policy information about cell lines and Sex and Gender in Research

Cell line source(s): ATTC: HCT116 cells (CCL-247) , U2OS cells (HTB-96)

Authentication: None of the cell lines were authenticated.

Mycoplasma contamination: All cell lines were tested and resulted negative for mycoplasma contamination

Commonly misidentified lines (See ICLAC register): No misidentified lines

Plants

Seed stocks: *Report on the source of all seed stocks or other plant material used. If applicable, state the seed stock centre and catalogue number. If plant specimens were collected from the field, describe the collection location, date and sampling procedures.*

Novel plant genotypes: *Describe the methods by which all novel plant genotypes were produced. This includes those generated by transgenic approaches, gene editing, chemical/radiation-based mutagenesis and hybridization. For transgenic lines, describe the transformation method, the number of independent lines analyzed and the generation upon which experiments were performed. For gene-edited lines, describe the editor used, the endogenous sequence targeted for editing, the targeting guide RNA sequence (if applicable) and how the editor was applied.*

Authentication: *Describe any authentication procedures for each seed stock used or novel genotype generated. Describe any experiments used to assess the effect of a mutation and, where applicable, how potential secondary effects (e.g. second site T-DNA insertions, mosiacism, off-target gene editing) were examined.*

ChIP-seq

Data deposition

☒ Confirm that both raw and final processed data have been deposited in a public database such as GEO.

☐ Confirm that you have deposited or provided access to graph files (e.g. BED files) for the called peaks.

Data access links (*May remain private before publication.*): GEO SuperSeries GSE173452

Files in database submission:
GSM6265475 WT1_S4
GSM6265476 WT1_S25
GSM6265477 MUT1_S7
GSM6265478 MUT1_S28
GSM6265479 WT2_S5
GSM6265480 WT2_S25
GSM6265481 MUT2_S7
GSM6265482 MUT2_S28

Genome browser session (e.g. UCSC): No longer applicable

Methodology

Replicates: 2

Sequencing depth: 20M

Antibodies: CDC45 11881 (Cell Signaling)

Peak calling parameters	Metagene plots for the CDC45-treated ChIP-seq were generated with deepTools. Specifically, coverage track signals, using the bigwig files and the indicated regions as inputs, were normalized by RPKM and binned per nucleotide in windows of the indicated width in each plot.
Data quality	FastQC tool was used to assess data quality
Software	ChIP-seq sequences were trimmed with Trimmomatic (v.0.38), aligned with Bowtie2 (v.2.3.4.2.) Duplicate reads were marked with Picard (v.2.18.17). BigWig files were generated with bedGraphtoBigWig tools.

Flow Cytometry

Plots

Confirm that:

☒ The axis labels state the marker and fluorochrome used (e.g. CD4-FITC).

☒ The axis scales are clearly visible. Include numbers along axes only for bottom left plot of group (a 'group' is an analysis of identical markers).

☒ All plots are contour plots with outliers or pseudocolor plots.

☒ A numerical value for number of cells or percentage (with statistics) is provided.

Methodology

Sample preparation	Cells were synchronized by double thymidine block and released. Cells were fixed in PBS 2% PFA for 15 minutes at room 50 temperature, centrifuged, and incubated in 2N HCl 0.5% Triton for 30 minutes at room temperature. Followed by 0.1 M Na2B4O7 incubation, cells were treated with RNAse A, resuspended in PBS, and the DNA stained with propidium iodide 1 mg/mL (Sigma).
Instrument	Flow cytometer FACSCalibur
Software	DNA staining was recorded by BD CellQuest program. Cell cycle profiles were determined by considering the amount of labelled DNA (FL2-H) per cell.
Cell population abundance	Only dead cells and doublets were discarded from the analysis
Gating strategy	Synchronized cells at different time points were gated with PI, being PI low cells resident G1/G0 phase cells; PI middle cells resident S phase cell cells; PI high cells resident M phase cell cells.

☒ Tick this box to confirm that a figure exemplifying the gating strategy is provided in the Supplementary Information.
